# Therapeutic peptides: current applications and future directions

**DOI:** 10.1038/s41392-022-00904-4

**Published:** 2022-02-14

**Authors:** Lei Wang, Nanxi Wang, Wenping Zhang, Xurui Cheng, Zhibin Yan, Gang Shao, Xi Wang, Rui Wang, Caiyun Fu

**Affiliations:** 1 Zhejiang Provincial Key Laboratory of Silkworm Bioreactor and Biomedicine, College of Life Sciences and Medicine, Zhejiang Sci-Tech University, 310018 Hangzhou, China; 2 State Key Laboratory Cultivation Base for TCM Quality and Efficacy, Nanjing University of Chinese Medicine, Nanjing, 210046 China; 3 Department of Oncology, No.903 Hospital of PLA Joint Logistic Support Force, Xi Hu Affiliated Hospital of Hangzhou Medical College, Hangzhou, 310013 China; 4 Key Laboratory of Preclinical Study for New Drugs of Gansu Province, School of Basic Medical Sciences & Research Unit of Peptide Science, Chinese Academy of Medical Sciences, 2019RU066, Lanzhou University, 730000 Lanzhou, China; 5 Institute of Materia Medica, Chinese Academy of Medical Sciences and Peking Union Medical College, 100050 Beijing, China

**Keywords:** Drug development, Drug screening

## Abstract

Peptide drug development has made great progress in the last decade thanks to new production, modification, and analytic technologies. Peptides have been produced and modified using both chemical and biological methods, together with novel design and delivery strategies, which have helped to overcome the inherent drawbacks of peptides and have allowed the continued advancement of this field. A wide variety of natural and modified peptides have been obtained and studied, covering multiple therapeutic areas. This review summarizes the efforts and achievements in peptide drug discovery, production, and modification, and their current applications. We also discuss the value and challenges associated with future developments in therapeutic peptides.

## Introduction

Therapeutic peptides are a unique class of pharmaceutical agents composed of a series of well-ordered amino acids, usually with molecular weights of 500-5000 Da^1^. Research into therapeutic peptides started with fundamental studies of natural human hormones, including insulin, oxytocin, vasopressin, and gonadotropin-releasing hormone (GnRH), and their specific physiological activities in the human body^2^. Since the synthesis of the first therapeutic peptide, insulin, in 1921, remarkable achievements have been made resulting in the approval of more than 80 peptide drugs worldwide. The development of peptide drugs has thus become one of the hottest topics in pharmaceutical research.

The first half of the 20th century witnessed the discovery of several life-saving bioactive peptides, such as insulin and adrenocorticotrophic hormone, which were initially studied and isolated from natural sources. The discovery and development of insulin, a peptide with 51 amino acids, has been considered as one of the monumental scientific achievements in drug discovery. It was first isolated by Frederick Banting in 1921 and further developed by Frederick and Charles Best^3,4^, and was already available for patients with diabetes mellitus just a year after its first isolation. In 1923, insulin became the first commercial peptide drug and has since benefited thousands of diabetes patients to date. However, the production of human insulin during the 20th century could not keep up with the high market demand, and animal-derived insulins, such as bovine and porcine insulin, dominated the insulin market for almost 90 years until they were replaced by recombinant insulin^5,6^.

More peptide hormones and their receptors with therapeutic potential were identified and characterized from the 1950s to the 1990s^7^. Meanwhile, the technologies used for protein purification and synthesis, structure elucidation, and sequencing made substantial progress, thus accelerating the development of peptide drugs, leading to nearly 40 peptide drugs being approved worldwide. Notably, synthetic peptides such as synthetic oxytocin^8^, synthetic vasopressin^9^, and recombinant human insulin^10,11^ began to be developed in addition to natural peptides.

Peptide drug development entered a new era with the advent of the 21st century, since when advances in structural biology, recombinant biologics, and new synthetic and analytic technologies have significantly accelerated the process. A sophisticated system of peptide drug development has been established, including peptide drug discovery, drug design, peptide synthesis, structural modification, and activity evaluation. A total of 33 non-insulin peptide drugs have been approved worldwide since 2000 (Table 1). In addition, these peptide drugs are no longer simply hormone mimics or composed simply of natural amino acids. For example, enfuvirtide is a 36-amino acid biomimetic peptide mimicking human immunodeficiency virus (HIV) proteins used in combination therapy for the treatment of HIV-1^12,13^; ziconotide^14,15^ is a neurotoxic peptide derived from the cone snail *Conus magus*, which was approved in 2004 and is used to manage severe chronic pain; teduglutide is a glucagon-like peptide 2 (GLP-2) analogue used to treat short bowel syndrome^16,17^, and is manufactured using a strain of *Escherichia coli* modified by recombinant DNA technology; and liraglutide is a chemically synthesized analogue of human glucagon-like peptide 1(GLP-1)^18,19^, made by attaching a C-16 fatty acid (palmitic acid) with a glutamic acid spacer on lysine residue (position 26 in the sequence), which acts as a GLP-1 receptor agonist to manage type 2 diabetes mellitus (T2DM). All these peptide drugs have been used in a wide range of therapeutic areas, such as urology, respiratory, pain, oncology, metabolic, cardiovascular, and antimicrobial applications^20–24^. To date, more than 170 peptides are in active clinical development (Table 2), with many more in preclinical studies^1,7^.

**Table 1:** Peptide drugs approved since 2000, with their targets and indications

Target name	Peptide name	First approval	Approved indication(s)
GLP-1 receptor	Exenatide^462^	2005	Indicated for Type 2 Diabetes Mellitus
	Liraglutide^463^	2009	
	Lixisenatide^464^	2013	
	Albiglutide^465^	2014	
	Dulaglutide^466^	2014	
	Semaglutide^467^	2017	
GLP-2 receptor	Teduglutide^468^	2012	Treatment of Short bowel syndrome and malabsorption
GC-C receptor	Linaclotide^469^	2012	Treatment of irritable bowel syndrome (IBS) with constipation and chronic idiopathic constipation
Calcitonin receptor	Pramlintide^470^	2005	Treatment of Type 1 and Type 2 Diabetes Mellitus
GnRH receptor	Abarelix^471^	2003	Treatment of advanced prostate cancer
	Degarelix^472^	2008	
Binding to active site of the 20S proteasome	Carfilzomib^473^	2012	Treatment of multiple myeloma
NOD2 protein	Mifamurtide^474^	2009	Treatment of high-grade, resectable, non-metastatic osteosarcoma
VIP1 receptor	Aviptadil^475^	2000	Treatment of erectile dysfunction
OT receptor	Atosiban^476^	2000	Indicated for use in delaying imminent pre-term birth
	Carbetocin^476^	2001	Used for postpartum hemorrhage
TRH receptor	Taltirelin^477^	2000	Spinocerebellar degeneration
MC receptors	Bremelanotide^478^	2019	Indicated for hypoactive sexual desire disorder
PTH1 receptor	Teriparatide^479^	2002	Treatment of osteoporosis
	Abaloparatide^480^	2017	
Guanylate cyclase C	Plecanatide^481^	2017	Treatment of chronic idiopathic constipation
NPR-A	Nesiritide^482^	2001	Treatment of acute decompensated heart failure
AT_1_ receptor	Angiotensin II^483^	2017	Indicated for sepsis and septic Shock
Beta2-receptor	Icatibant^484^	2008	Approved for use in acute attacks of hereditary angioedema
gp41	Enfuvirtide^485^	2003	Used in combination therapy for the treatment of HIV-1
GHRH receptor	Tesamorelin^486^	2010	Reduction of HIV lipodystrophy
N-type calcium channels	Ziconotide^487^	2004	Management of severe chronic pain
Thrombopoietin receptor	Romiplostim^488^	2008	Treatment of chronic immune thrombocytopenic purpura
Human erythropoietin receptor	Peginesatide^489^	2012	Treatment of anemia associated with chronic kidney disease
Pulmonary surfactant	Lucinactant^490^	2012	Prevention of respiratory distress syndrome
CaSR	Etelcalcetide^491^	2016	Indicated for secondary hyperparathyroidism
MC1 receptor	Afamelanotide^492^	2014	Prevention of phototoxicity
Somatostatin receptors	Pasireotide^493^	2012	Treatment of Cushing’s disease
	Lutetium Lu 177 dotatate^494,495^	2018	Treatment of somatostatin receptor-positive gastroenteropancreatic neuroendocrine tumors
	Edotreotide gallium Ga-68^496,497^	2019	Indicated for diagnose somatostatin receptor positive neuroendocrine tumors
Melanocortin-4 receptor	Setmelanotide^498,499^	2020	Indicated for chronic weight management of obesity

**Table 2:** Examples of peptides in different clinical trials and their indications

Clinical trial phase	Peptide name	Indication(s) for investigation
IV	Avexitide^500^	Hypoglycemia
	Calcitonin gene-related peptide^501^	Migraine
	Corticorelin^502^	Brain swelling; brain neoplasms
	Leptin^503^	Lipodystrophy; obesity
	Thymalfasin^504^	Liver Cirrhosis; Sepsis
III	Aclerastide^505,506^	Diabetic foot ulcers
	Albusomatropin^507^	Growth hormone deficiency
	Anamorelin^508^	Cachexia; lung cancer non-small cell cancer
	G17DT^509^	Various forms of cancer
	Insulin peglispro^510^	Diabetes mellitus
	Lenomorelin^511^	Malignancies
	Selepressin^512^	Shock; septic
	Somapacitan^513^	Adult growth hormone deficiency
	Taspoglutide^514^	Type 2 diabetes mellitus
	Thymosin beta-4^515^	Dry eye syndrome
	Tirzepatide^516^	Type 2 diabetes mellitus
	Ularitide^517^	Decompensated heart failure
	Vapreotide^518^	Gastric varices; oesophageal haemorrhage; portal hypertension; esophageal varices
	Vosoritide^519^	Achondroplasia
	Zoptarelin doxorubicin^520^	Endometrial cancer; prostate cancer
II	Angiotensin 1-7^521^	Miscellaneous Peripheral Blood Cell Abnormalities
	Bombesin^522^	Prostate cancer
	Cenderitide^310^	Heart failure
	Deslorelin^523^	Puberty; precocious
	Gastric inhibitory polypeptide^524^	Type 2 diabetes mellitus
	MK-3207^525^	Migraine
	Olcegepant^526^	Migraine Disorders
	Pancreatic Polypeptide^527^	Type 1 diabetes
	Peptide YY (3-36)^528^	Metabolic disease; obesity
	Pirnabine^529^	Chronic idiopathic constipation
	Somatoprim^530^	Acromegaly
	Somatropin pegol^531^	Growth hormone deficiency
	Thyrotropin^532^	Benign nontoxic and toxic goiter; goiter; nodular
	TT-232^533^	Renal cell adenocarcinoma
I	BPI-3016^534^	Type 2 diabetes mellitus
	NBI-6024^535^	Type 1 diabetes mellitus
	Many more…	

**Fig. 1:**
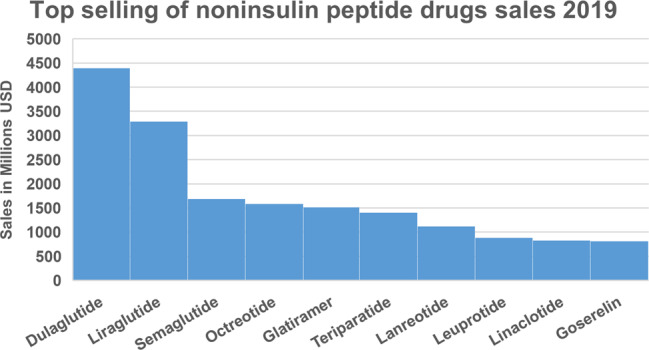
Top-selling non-insulin peptides worldwide in 2019. Data analysis according to Njardarson’s group^27^

Peptide drugs account for a significant proportion of the pharmaceutical market, with worldwide sales of more than $70 billion in 2019^25^, a more than two-fold increase compared with 2013^26^. According to Njardarson et al., the top 200 drug sales in 2019^27^, included 10 non-insulin peptide drugs. Interestingly, the top three sales of peptide drugs were all GLP-1 analogues for treating T2DM, including Trulicity (dulaglutide) ranked at 19 with $4.39 billion retail sales, Victoza (liraglutide), ranked at 32 with $3.29 billion sales, and Rybelsus (semaglutide), ranked at 83 with $1.68 billion sales (Fig. 1).

In this article, we review the historical development of peptide drugs and current advances in peptide drug discovery. We focus on the pharmaceutical characteristics of therapeutic peptides and highlight new technologies that have improved the design, synthesis, modification, and evaluation of peptide drugs, and provide new perspectives in the applications of peptide drugs. We also refer readers to several recent reviews for further reading^1,7,28^.

## Therapeutic peptides: advantages and drawbacks

Therapeutic peptides commonly act as hormones, growth factors, neurotransmitters, ion channel ligands, or anti-infective agents. They bind to cell surface receptors and trigger intracellular effects with high affinity and specificity, with a similar mode of action to biologics, including therapeutic proteins and antibodies. However, compared with biologics, therapeutic peptides show less immunogenicity and have lower production costs^29–32^.

Small molecule drugs are known to have an extended therapeutic history with inherent advantages, including low production costs and sale prices, oral administration, and good membrane penetration ability^33^. Both naturally extracted and chemically synthesized small molecules show competitive price advantages compared with peptides and biologics (proteins or antibodies)^34,35^. Oral administration of small molecules has the benefits of better safety and improved patient compliance, while their small size also enables them to penetrate the cell membrane to target intracellular molecules^33,36^. However, their small size also means that it is difficult for them to inhibit large surface interactions, such as protein-protein interactions (PPIs), effectively. PPIs usually occupy a contact area of 1500–3000 A^2^, while small molecules only cover 300–1000 A^2^ of the protein surface, due to their limited molecular size^37^. By contrast, the unique physiochemical properties of peptide drugs, including their larger size and more flexible backbone, enable them to act as potent inhibitors of PPIs^38^. The clinical use of small molecules is also limited by their low specificity compared with peptide drugs. For example, sorafenib and sunitinib are tyrosine kinase inhibitors that inhibit the tyrosine kinase domain activity of vascular endothelial growth factor (VEGF) receptors, resulting in anti-angiogenic effects that are used to treat cancer patients^39–41^; however, they also target other kinase receptors such as serine/threonine kinase receptors, leading to cytotoxicity^42–46^.

**Fig. 2:**
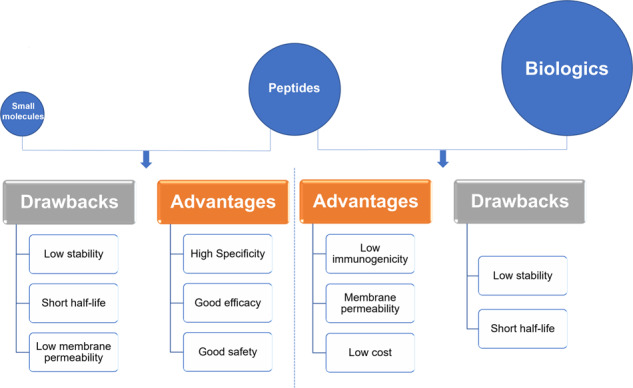
Peptides versus small molecules and biologics. Comparison of advantages and drawbacks between peptides and small molecules or biologics

As natural amino acid-based therapeutics, therapeutic peptides have two intrinsic drawbacks (Fig. 2): membrane impermeability and poor in vivo stability, which represent major stumbling blocks for peptide drug development^2,29^.

1. Peptides have weak membrane permeability. The membrane permeability of peptide drugs depends on multiple factors, including peptide length and amino acid composition. Peptides are generally unable to cross the cell membrane to target intracellular targets, thus limiting their applications in drug development. Lau et al. reported in 2018 that >90% of peptides in active clinical development targeted extracellular targets, including G-protein coupled receptors (GPCRs), gonadotropin-releasing hormone (GnRH) receptor, Glucagon-like peptide 1 (GLP-1) receptor^7^.
2. Peptides have poor in vivo stability. Natural peptides consist of chains of amino acids joined by amide bonds, but lack the stability conferred by secondary or tertiary structures. The amide bonds can be easily hydrolyzed or destroyed by enzymes in vivo, upon exposure to the environment, without any protection. These inherent chemical properties make the peptides chemically and physically unstable, with a short half-life and fast elimination in vivo^47^.

These intrinsic advantages and disadvantages of peptides present both challenges in peptide drug development and also opportunities and directions for peptide drug design and optimization.

## Developmental path of therapeutic peptides: discovery, production, and optimization

### Peptide drug discovery

#### Natural peptides/hormones in the human body

**Fig. 3:**
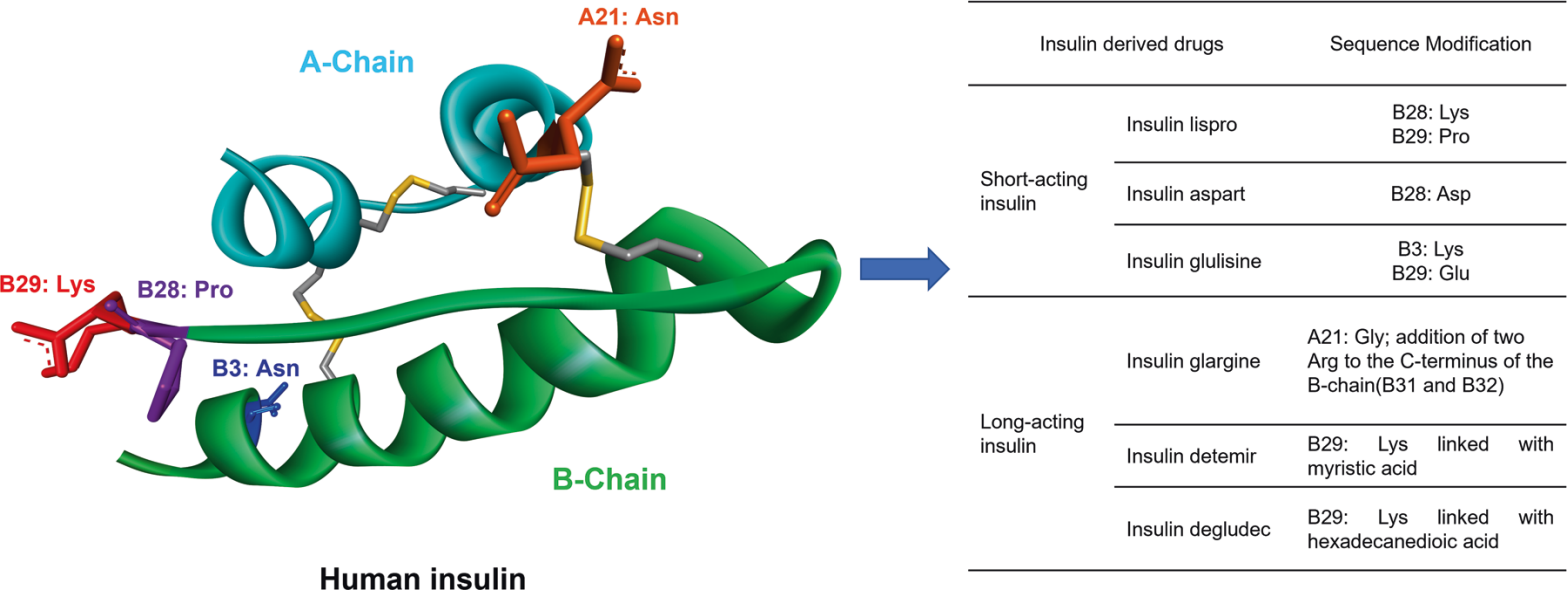
Structure of human insulin and human insulin-derived drugs. Structure of human insulin (left, PDB: 1XDA). Modifications on its residues (B-Chain: B3: Asn, B28: Pro, B29: Lys; A-Chain: A21: Asn) resulted in several short- and long-acting insulin drugs (right, see table)

The history of peptide drug discovery started by exploiting natural hormones and peptides with well-studied physiological functions for treating diseases caused by hormone deficiencies, such as a lack of insulin required to regulate blood glucose levels in patients with T1DM or T2DM. Diabetes is treated either by insulin injection or by stimulating insulin secretion-related targets such as GLP-1 receptor, to produce insulin^48^. Searching for natural peptides and hormones or replace them by animal homologues, such as insulin, GLP-1, somatostatin, GnRH, 8-Arg-Vasopressin, and oxytocin, were the initial strategies used for peptide drug discovery and development (Fig. 3). However, the drawbacks associated with these natural peptides aroused interest in optimizing their natural sequences, leading to a series of natural hormone-mimetic peptide drugs.

#### Peptides mimicking hormones

**Fig. 4:**
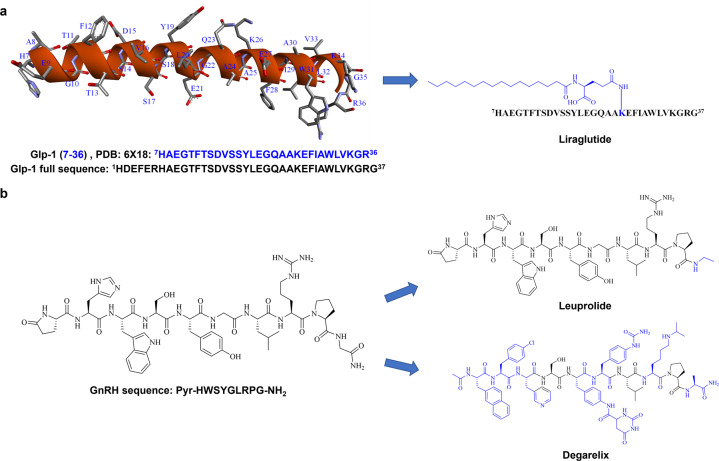
Sequences and structures of natural hormones GLP-1 and GnRH and their peptidomimetic drugs. **a** Liraglutide is a GLP-1 derived peptide drug, modified on 26^th^ residue (K) of its natural sequence. **b** Leuprolide and degarelix are modified from the natural sequence of GnRH

GLP-1 derived peptide drugs (Fig. 4a): GLP-1 is a 37-amino acid peptide that regulates insulin production and secretion^49^, with a very short half-life in vivo. Extensive efforts have been made to modify its sequence to enhance the stability of this hormone, while maintaining its potency and pharmacological effect^50,51^, leading to the development of the three top-selling anti-T2DM peptide drugs: Trulicity (dulaglutide), Victoza (liraglutide), and Ozempic (semaglutide).

Gonadotropin-releasing hormone (GnRH) derived peptide drugs (Fig. 4b): GnRH is a peptide containing 10 amino acids that is produced by GnRH neurons in the hypothalamus^52^. Modification of the native sequence of GnRH has led to the development of several peptide drugs, such as leuprolide and degarelix. Leuprolide has the same biological activity as GnRH by activating GnRH receptors, and is used as a GnRH receptor agonist for treating hormone-responsive prostate cancer, endometriosis, uterine fibroids, and precocious puberty^53,54^. While the sequence of degarelix is optimized from GnRH, it acts as a GnRH antagonist by competitively binding to the GnRH receptor and is used to treat terminal prostate cancer^55^.

Many other approved peptide drugs are also derived from natural hormones^1^, including octreotide, a somatostatin mimic peptide drug, used for the treatment of growth hormone producing tumors and pituitary tumors^56,57^; desmopressin, an 8-Arg-vasopressin mimicking peptide drug, used for diabetes insipidus and nocturia^58^; carbetocin, an oxytocin homologue used to treat amenorrhea^59^ and atosiban, an oxytocin antagonist for suppressing premature labor^60^.

#### Peptides identified from natural products

**Fig. 5:**
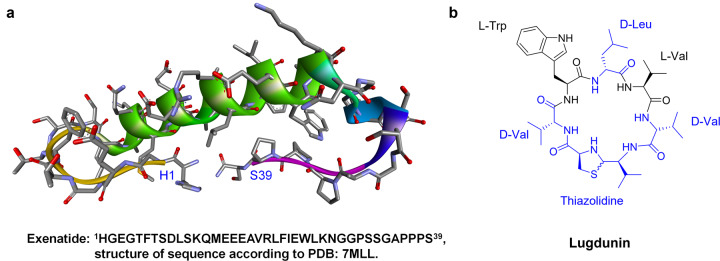
Sequences and structures. Exenatide (**a**) and lugdunin (**b**)

Many bioactive peptides from bacteria, fungi, plants, and animals possess therapeutic properties, such as snake venom, which is considered as a vascular endothelial growth factor (VEGF) analogue, VEGF-F or svVEGF^61–63^. They are usually disulfide-rich cyclic peptides of no more than 80 residues, which can induce cytotoxicity by targeting ion channels and other membrane-bound receptors^1,64^. Venom peptides from snakes and scorpions have been modified for therapeutic applications. In addition, exenatide (Fig. 5a), optimized from Gila monster venom^65^ is a GLP-1 agonist and ziconotide, a venom peptide derived from *Conus magus*, has been used to treat chronic neuropathic pain^66,67^.

Non-ribosomal peptides (NRPs) comprise another class of peptides identified from natural products. The non-standard residues contained in the sequence mean that NRPs are not produced through the traditional biosynthesis pathways via ribosomes^68^, but are produced by non-ribosomal peptide synthetases via a pathway consisting of initiation, elongation, and termination modules^69,70^. Compared with peptides synthesized by ribosomes, NRPs are more resistant to hydrolases and show increased stability in vivo. The most-studied NRPs are mainly derived from bacteria and fungi, including vancomycin, cyclosporin, lugdunin^71,72^. (Fig. 5b), and teixobactin with antibacterial activities, and a-amanitin, nanocystin A, and actinomycin with anti-tumor activities^73,74^. In addition, cyclodepsipeptides are cyclic peptides that comprise a specific class of NRPs usually identified in plants^75–77^, such as enniatin B and emodepside^78,79^. These peptide drugs display enhanced plasma stability that enables their oral delivery. However, the synthesis and structure-activity relationships study of NRPs represent one of the most challenging and exciting areas of research for NRPs.

#### Rational design of peptides based on Protein–Protein Interactions

Developments in proteomics and structural biology have led to the discovery of many Protein-Protein interactions (PPIs) involved in most cellular processes and biological functions^80,81^. Over 14,000 PPIs, accounting for only about 1% of all PPIs in the human body, have been studied to date^82^. PPIs also regulate many essential cellular pathways in human diseases and are thus potential drug targets^83^. Peptides contain intrinsic advantages as inhibitors or activators of PPIs compared with small molecules and antibodies. Therefore, a new peptide drug discovery technology based on the known crystal structure of PPIs has thus been developed: the rational design of peptides. It is considered to be a promising strategy for the discovery of new peptide drug candidates^84,85^.

The rational design of peptides involves computer-assisted bioinformatics technology based on the resolved crystal structure of the target PPIs. Bioinformatic and computational analysis of the PPI binding interface enables the essential amino acids on the surface of the two interacting proteins to be identified. These essential amino acids contribute the major Gibbs energy of the PPIs and are commonly called “hotspots”^86,87^. Hotspots may be a continuous fragment of the protein or dispersed residues on different secondary structures of the protein. The design of peptide modulators for PPIs is based on these hotspots, either directly using the continuous fragment or using a strategy to link the dispersed residues as initial sequences^88^. However, further peptide development and structure optimization including peptide cyclization and backbone modification are required to improve their activity and physicochemical properties^89,90^. For example, identification of the essential peptide residues and the proposed substitution of non-essential residues via study of the structure-activity relationship, and chemical modification of the sequence to stabilize the peptide secondary structure, including turns, helices, hairpins, and extended conformations, can be applied to enhance the bioactivity and improve the physicochemical properties^91,92^.

#### Discovery of peptide drug candidates by phage display

Phage display is a highly effective and robust technology used to identify ligands of biological targets, first reported by Smith in 1985^93^. Phage display uses recombinant technologies to engineer target ligands on the surface of the bacteriophage^94^. Only peptides containing proteinogenic amino acids, rather than NRPs, are produced in the phage. This high-throughput sequencing method can be used to identify drug leads, including antibodies and peptides^95,96^. Phage display has been widely used to discover new peptide ligands. Lerner et al. reported the discovery of potent peptide analogues of GLP-1 and other membrane receptor ligands by phage display, including proteins, peptides, and venoms, which mainly act as agonists^97–100^. In addition, peptides targeting transforming growth factor (TGF)-β1^101^ or epidermal growth factor receptor (EGFR)^102^, and peptide antagonists that disrupt the fibroblast growth factor (FGF)-1-FGFR1 interaction^103^ are good examples of peptide drugs discovered by phage display. Recent developments in phage display technology have focused on searching for more efficient screening protocols to simplify ligand selection among enormous amounts of data, such as by reducing phage panning cycles^104^. Heinis et al. used an “on-phage” modification technology to obtain chemically modified peptides from traditional phage display to obtain a bis-thioether cyclic peptide^105^. Another strategy involves developing novel display approaches. For example, Schumacher et al. developed a mirror-image phage display to explore D-chirality peptides^106,107^, and Szostak et al. performed mRNA display to discover and select macrocyclic peptides with unnatural amino acids^108–110^. Suga et al. used ribosomal display to exploit lead peptides, including bioactive macrocyclic peptides, containing D-amino acids and unnatural amino acids^111–113^. These developments have allowed the construction of numerous display libraries for the discovery of new peptide candidates.

## Synthesis and modification of therapeutic peptides

The discovery of potential therapeutic peptides is the first step peptide drug development, followed by chemical or biological peptide synthesis and sequence modification to improve its pharmacological properties. Here we summarize the fundamental technologies utilized for peptide production and modification.

### Chemical synthesis of peptides

The chemical synthesis of peptides is well-developed, particularly solid-phase peptide synthesis (SPPS) technology developed by Merrifield in 1963^114^. SPPS technology has since been remarkably improved in terms of its methodology and synthetic materials and plays a crucial role in modern peptide production. It facilitates peptide synthesis by combining amino acid coupling and deprotection in one simple reactor, which has further led to the invention of automatic peptide synthesizers. Compared with recombinant technology, the crude peptides obtained by SPPS are more monotonous, without other biological compounds such as enzymes, DNA and RNA fragments, non-related proteins, and peptides. Moreover, the impurities in the final SPPS product are easily identified because they are mainly derived from incomplete or side reactions during the synthesis procedure^115^, making subsequent purification relatively uncomplicated^116^.

**Fig. 6:**
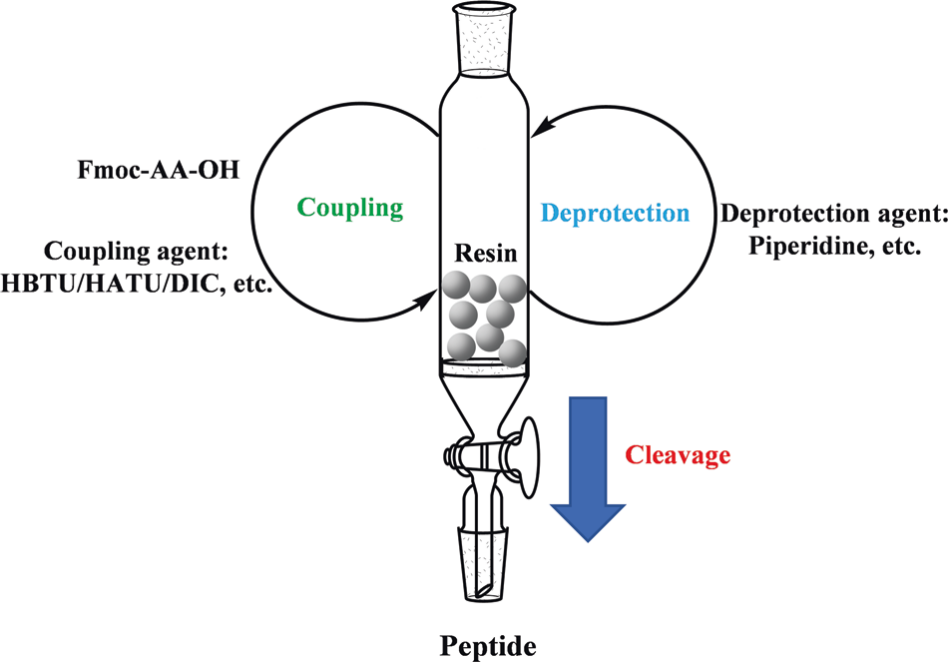
A general process of solid-phase peptide synthesis (SPPS) with Fmoc protected amino acids (Fmoc-AA-OH). Fmoc-SPPS consists a cycle of coupling Fmoc-AA-OH to a solid polymeric resin and deprotection of Fmoc to liberate amino groups. The whole process can be carried out in a sieve reactor till the final peptide is cleaved from the resin

SPPS consists of a cycle of coupling the carboxylic group of amino acids to a solid polymeric resin, and liberation of the amine group from the protection group (Fig. 6). Various resins, such as 4-methylbenzhydrylamine (HMBA) resin, Wang resin, 2-chlorotrityl chloride (CTC) resin, and Merrifield resin, are used to introduce either amide or free carboxylic groups into the C-terminal of peptide. The modern peptide industry has developed various functional resins by coupling the resins with different linkers, enabling the synthesis of long peptides and peptide cyclization in the solid phase^117^. During synthesis, the amine group of the amino acids and the side chains are usually protected by different chemical groups, which cause peptide aggregation and reduce the purity of the crude peptides. Two major SPPS strategies: Fmoc-SPPS and Boc-SPPS have been developed to remove the predominant amine protection groups, fluorenylmethyloxycarbonyl (Fmoc) and t-butyloxycarbonyl (Boc), respectively^118,119^.

Boc-SPPS uses trifluoroacetic acid solution to remove the amine protection groups and hydrogen fluoride solution to cleave the final peptide, but these processes are associated with irritating odor and toxicity. Fmoc can be removed under milder conditions, and the Fmoc-SPPS strategy is thus often preferred^120^. However, Boc-SPPS has advantages for long peptide synthesis, because trifluoroacetic acid deprotection effectively destroys the aggregation during the peptide synthesis^121^. Fmoc-SPPS research is currently focused on resolving two major problems, including aggregation during long peptide synthesis and the formation of aspartimides for certain sequences^118^. Multiple methods have been utilized, including applying low-substitution resins to separate peptide chains^122^, microwaves to reduce the reaction time^123^, mixed solvents as a reaction solution^124^, and pseudoprolines to break the H-bond of the backbone to avoid or reduce aggregation during SPPS^121^. Aspartimide formation during Fmoc-SPPS significantly decreases the purity of the crude peptides. The solutions applied to reduce aspartimide formation were using microwaves to reduce the reaction time^125^, or using N-α-alkyl Asp–Gly dipeptide^126^, or adding 1-hydroxybenzotriazole (HOBt)^127^, Oxyma Pure^128^ during the deprotection process.

The synthesis of peptides of <50 residues by Fmoc-SPPS is relatively routine, but the chemical synthesis of longer peptides (>50 amino acids) is still challenging, especially in large-scale manufacture. Laboratory-scale peptide synthesis tends to be carried out automatically with the help of modern automated peptide synthesizers, such as CEM Liberty PRIME and CSBio II. These new automatic peptide synthesizers can provide sequential and multi-parallel peptide syntheses of up to 192 different sequences, using infrared or microwave heating to reduce the reaction time, and sometimes using ultraviolet monitoring to ensure the deprotection process^129,130^. Such synthesizers are extremely helpful for laboratory-scale peptide synthesis, producing the desired peptides rapidly for further structural and functional studies. However, there are limited applications of infrared and microwave heating to large-scale peptide manufacture due to a lack of large equipment and nonhomogeneous overheating, which may lead to the production of byproducts^131^. Most good manufacturing practice (GMP) thus prefers mild reaction conditions to minimize side reactions and relative impurities, and the large-scale production of long peptides (>50 amino acids) thus remains challenging.

The development of chemical peptide synthesis, especially by SPPS, has significantly accelerated the development of therapeutic peptides. Some recombinant peptide drugs, such as oxytocin and teriparatide, use chemical synthesis to produce active pharmaceutical ingredients. The chemical synthesis of peptides also permits their kaleidoscopic modification.

### Chemical modification of peptide and peptidomimetics

As a particular class of therapeutic agents, the biological activity of peptides is intimately related to their chemical structure. Following the synthesis of peptides, they need to be modified using medicinal chemistry techniques to mimic, stabilize, or construct an ideal secondary structure to improve their biological activity and achieve selectivity, stability, and solubility of the peptide drugs^132^.

Before modification of the lead peptide drug candidate, it is necessary to identify the minimum active sequence with the desired biological properties. Classical sequence scanning, termed alanine-scan^29,133,134^, is then commonly used to replace each residue with alanine to produce a series of lead peptide analogues to determine which key residues confer the biological activity of the lead peptide: a decrease in activity suggests that the replaced residue was important, whereas a non-significant reduction of activity suggests that the replaced residue was redundant. Further modifications of the replaceable residues and C- and N-termini of the lead peptide are then carried out to produce the final peptide drug^135^.

#### Backbone modification of peptides

One of the main reasons for backbone modification is to improve the proteolytic stability of the peptide. Proteolytic sites in the peptide can be identified by stability studies and metabolite determination^136^. Backbone modification includes the substitution of L-amino acids by D-amino acids^137,138^, insertion of methyl-amino acids^137,139^, and the incorporation of β-amino acids^140^ and peptoids^141–143^. Introducing these non-natural amino acids into the peptide sequence, particularly at the proteolysis site, is an effective strategy for extending the plasma half-life of peptide drugs. A successful example is selepressin, which was derived from vasopressin and has similar target selectivity but a longer plasma half-life^144,145^.

#### Side chain modification of peptides

Side chain modification of peptides is achieved by replacing the natural amino acids with their analogues during peptide synthesis, to improve their binding affinity and target selectivity^1,146^. Variants of natural amino acid analogues such as homoarginine, benzyloxy-tyrosine, and β-phenylalanine are commonly commercially available^147^, and can be conveniently used to chemically modify the peptide side chain during peptide synthesis^148^. Several GLP-1 analogue drugs such as liraglutide and semaglutide have modified side chains^48^.

#### Mimicking and stabilization of secondary structures by backbone and side chain modification

The weak forces in peptides, such as hydrogen bonds, van der Waals forces, and intramolecular hydrophobic interactions are not adequate for a stable secondary structure conformation. Additional modifications of the backbone, N- or C-termini, or side-chains to mimic the structures of natural products or hot spots in PPI and stabilization of secondary structures are therefore needed to produce promising peptide drug candidates^149,150^.

**Fig. 7:**
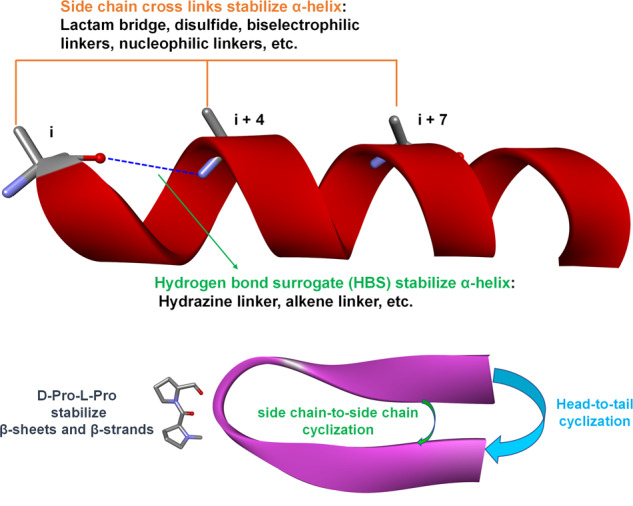
Strategies of peptide cyclization and stabilization of α-helices, β-sheets, and β-strands. The establishment of intramolecular cross-links can stabilize different secondary structures of peptides. Side chain cross-links between i and i + 4 or/and i + 7 and hydrogen bond surrogate cross-links can stabilize α-helices. Side chain-to-side chain, head-to-tail, and side chain-to-tail cyclization can stabilize turn, loop and β structures (β-sheets and β-strands). The D-Pro-L-Pro scaffold can specifically stabilize antiparallel β-hairpins
- **Peptide mimicking of α-helices and stabilization**. Helices are one of the commonest types of protein secondary structures, representing about 30%-40% of all protein structures^164^. The α-helix is formed by intramolecular hydrogen bonds^165^ and accounts for 90% of helix structures^165^. Mimicking the α-helix in peptides enables the identification of modulators of PPIs. The α-helix can be stabilized either by building cross-links through side chains or replacing hydrogen bonds by covalent bonds (referred as hydrogen bond surrogates, HBS). In the α-helix structure, the side chains of amino acids at positions i, i + 4, and i +7 are on the same side, and building cross-links through i and i + 4 or i and i +7 effectively approach backbone atoms and help to form hydrogen bonds in helical structures^166–168^. Great efforts have been made to investigate different cross-links, such as lactam-based cross-links (Fig. 7), with the formation of a lactam bridge through the side chain of glutamic acid or aspartic acid with lysine^169^, the formation of disulfide bonds by replacing residues with cysteine or homocysteine^170^, and biselectrophilic linkers^171,172^. Stapled peptides represent a recent new cross-linking approach introduced to stabilize the α-helix structure, using non-natural electrophilic amino acids to replace residues at the i and i + 4 or i and i +7 position, and form ligations with nucleophilic cross-links^154,173,174^. The HBS modification strategy involves replacing one hydrogen bond of the α-helix peptide with a covalent bond to pre-organize the helical structure. Cabezas and Satterthwait first used hydrazine links to build an HBS peptide to mimic an α-helix^175^. The Arora group has also carried out extensive work on HBS peptides, using alkene linkers to stabilize the α-helix^176–179^. They recently started to use the HBS strategy to stabilize β-hairpins^180–182^, as well as the biological activities of these modified peptides^181,183–185^. We also used the HBS peptide modification strategy in our previous work, focusing on designing a full SPPS pathway to simplify the application of HBS in α-helix mimicking and stabilization^186,187^.
- **Peptide mimicking of β-strands and β-sheets**. β-sheets and β-strands represent another class of protein secondary structures, based on turn mimics. The modification of peptides to stabilize β-sheets is usually achieved by the introduction of D-amino acids, such as D-Pro, to form a turn structure in the sequence. D-Pro-L-Pro templates are a well-known scaffold for stabilizing antiparallel β-hairpins in several successful PPI inhibitors^188,189^. Macrocyclization or amyloid beta-sheet mimics have also been applied to create β-sheets and β-strand structures^190–193^.

- **Peptide cyclization**. Cyclization is a common peptide modification technique that can include various strategies, such as head-to-tail, backbone-to-side chain, and side chain-to-side chain cyclization (Fig. 7)^151–153^. Peptide cyclization can increase proteolytic stability^154,155^, and cell-permeability^156–158^, and allows mimicking and stabilization of the peptide secondary structure. Without being connected to other peptides, a single peptide sequence cannot form loop or turn structures, but cyclization facilitates the formation of these secondary structures by pre-organizing intramolecular interactions^159,160^. Peptide cyclization is also commonly applied to stabilize other secondary structures, such as α-helixes and β-sheets^161–163^.

Chemical modification is an effective method of producing peptide analogues with the desired structures. The improved stability and activity have resulted in the introduction of several peptide drugs into the clinic, such as selepressin, liraglutide, and semaglutide. However, some modifications cannot improve the proteolytic stability and activity simultaneously. For example, the insertion of D-amino acid can usually help to extend the plasma half-life of the peptide, but peptides with D-amino acid modification rarely exhibit effective biological activity^1,137,194^.

### Peptide production by recombinant technology

Chemical synthesis is the preferred method for the industrial preparation of peptides, because it can introduce versatile synthetic building blocks beyond the proteinogenic amino acids, such as unnatural amino acids, and biochemical or biophysical probes, allowing further modification or conjugation. Furthermore, the chemical synthesis process can be fully automated and easily scaled up. It provides a convenient and efficient approach for producing short- and medium-sized peptides, but the chemical synthesis of long peptides remains challenging, and alternative strategies are therefore required.

In addition to chemical synthesis, therapeutic peptides can be prepared by various biological methods, such as isolating bioactive peptides from natural sources by extraction^195^, enzymatic synthesis^196^, fermentation^197,198^, recombinant DNA technology^199,200^, and semisynthesis^201,202^. These approaches can be applied exclusively or in combination, depending on the complexity and difficulty of preparing the peptide^203,204^.

The practice of isolating peptide drugs from natural sources can be traced back to the 1920s, when insulin was first isolated from livestock pancreata and used to treat diabetes^205,206^, saving hundreds of thousands of lives. The pioneering success of insulin led to increasing public enthusiasm for peptide therapeutics, and several other animal-derived peptide drugs subsequently successfully entered clinical use, such as adrenocorticotropic hormone^207^ and calcitonin^208^. Non-ribosomally synthesized peptides represent another important family of natural sources for identifying and producing peptides with therapeutic potential, as exemplified by vancomycin and cyclosporin. Unlike ribosomally synthesized peptides or proteins, the synthesis of non-ribosomally synthesized peptides is controlled by clusters of genes encoding non-ribosomal peptide synthetases rather than the endogenous translational machinery, leading to the production of structurally and functionally diverse peptides, and allowing these molecules to overcome the inherent limitations of common peptide drugs. Venoms and toxins are recognized as valuable natural sources as starting points for identifying bioactive peptides^208–210^, and other natural sources, such as cyclotides and lantipeptides have also been studied and exploited^211–213^. Enzymatic synthesis is suitable for the synthesis of short peptides, such as dipeptides and tripeptides, and enzymatically synthesized peptides have been successfully applied for the production of food additives, pharmaceuticals, and agrochemicals. Fermentation has been well-documented as an eco-friendly approach for producing bioactive peptides, such as in the manufacture of cyclosporine^214^. Recombinant DNA technology enables the production of peptides and proteins with defined sequences and homogeneity. This approach is particularly useful for manufacturing long or complicated peptides with multiple disulfide bonds, which can otherwise be difficult to synthesize chemically. Human insulin and growth hormone are representative examples of the many available peptide drugs made using recombinant DNA technology. In addition, recombinant DNA technology can be combined with genetic code expansion and other novel technologies to introduce desired functional groups into the molecules via the incorporation of unnatural amino acids, as discussed below. Semi-synthesis provides a flexible approach for producing large bioactive polypeptides by linking synthetic peptides and recombinant DNA-expressed peptides^215–217^, and is a particularly useful approach when multiple artificial modifications are needed.

### Peptides modification by genetic code expansion

**Fig. 8:**
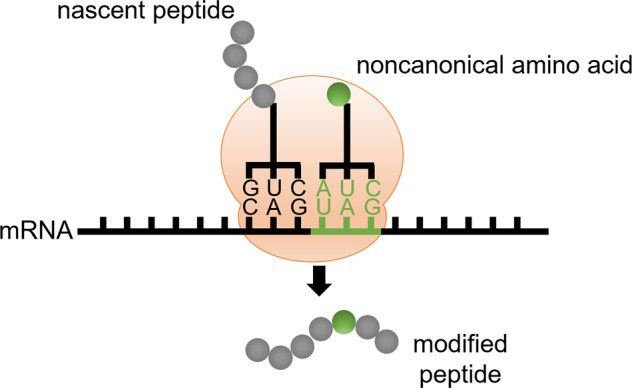
Scheme of genetic code expansion. Genetic code expansion enables the site-specific incorporation of an noncanonical amino acid (shown in green filled circle) into a growing peptide chain by suppressing an unique codon (e.g., amber stop codon)

Natural proteins are synthesized from 20 canonical amino acids, and this limited and conservative repertoire of amino acids significantly restricts the diversity and complexity of protein structures and functions. Genetic code expansion was developed two decades ago as a technology to overcome this limitation (Fig. 8)^218,219^. Genetic code expansion allows for the site-specific incorporation of non-canonical amino acids (ncAAs) with novel chemical and physical properties into a growing polypeptide during protein translation^220,221^. Four components are required to achieve this: 1) an ncAA with the desired chemical and physical properties; 2) a unique codon that specifies the ncAA, e.g., an amber stop codon (UAG) or quadruplet codon; 3) an orthogonal tRNA that suppresses the unique codon and does not crosstalk with its endogenous counterparts; and 4) an orthogonal amino-acyl tRNA synthetase that can specifically charge the ncAA onto the orthogonal tRNA and does not crosstalk with the endogenous amino-acyl tRNA synthetase/tRNA pairs^218,222–224^.

More than 200 ncAAs with diverse functionalities have been genetically encoded into different organisms to date, such as *Escherichia coli*, yeast, mammalian cells, viruses, and even animals, providing an invaluable toolbox for protein studies and engineering^225–232^. This expanded set of building blocks, including bioorthogonal chemical conjugation partners, metal chelators, photo-crosslinkers, proximity-enabled crosslinkers, photocaged amino acids, amino acids with post-translational modifications (phosphorylation, sulfation, acylation, etc.), redox-active amino acids, and infra-red, nuclear magnetic resonance, fluorescent probes, has been widely used in the study, manipulation, and evolution of proteins^233–242^. The ability to genetically encode diverse ncAAs allows for the rational optimization and production of chemically modified recombinant proteins with defined structures, functions, and stoichiometries^243,244^. Here, we focus on the application of genetic code expansion in the evolution of therapeutic peptides and proteins.

### PEGylation of peptides and proteins

Short protein and peptide therapeutics produced by genetic code expansion also have a short half-life because of their poor pharmacokinetics, including fast serum degradation and quick elimination. Attaching a polymer is one approach for extending the half-life of protein therapeutics^245^. PEG is formed by repetitive units of ethylene oxide and is a non-biodegradable, non-toxic, low-immunogenic polymer. PEGylation can increase the effective molecular weight of proteins to reduce their renal clearance by kidney filtration. The PEG moiety can also shield the proteins from digestion by proteolytic enzymes via increased steric hindrance, and help increase absorption by increasing the target protein’s water solubility^246^. These advantages make PEGylation a prevalent strategy for modifying therapeutic proteins, and PEGylation has been applied for optimizing protein therapeutics since the 1970s, with great success. There are currently >10 PEGylated protein therapeutics in the market, with more potential candidates in clinical trials^247^.

**Fig. 9:**
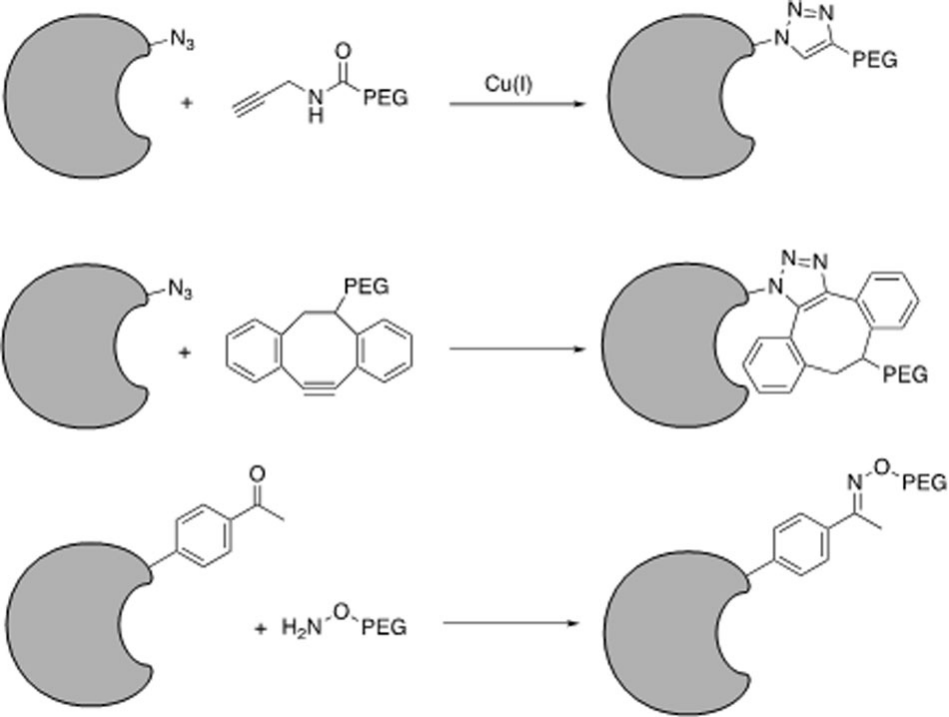
PEGylation of therapeutic peptides and proteins via genetic code expansion. Azide or acetyl groups are introduced into therapeutic peptides and proteins by genetic code expansion to allow downstream PEGylation modifications

Conventional PEGylation often occurs at Lys or Cys residues^248^. However, if the target protein includes more than one reactive Lys or Cys residue, conjugation can occur randomly at any of these residues due to a lack of selectivity, leading to the generation of heterogeneous conjugation products that are hard to separate. Techniques allowing site-specific PEGylation in which the PEG moieties can be attached to proteins with selectivity and positional control are thus needed (Fig. 9).

**Fig. 10:**
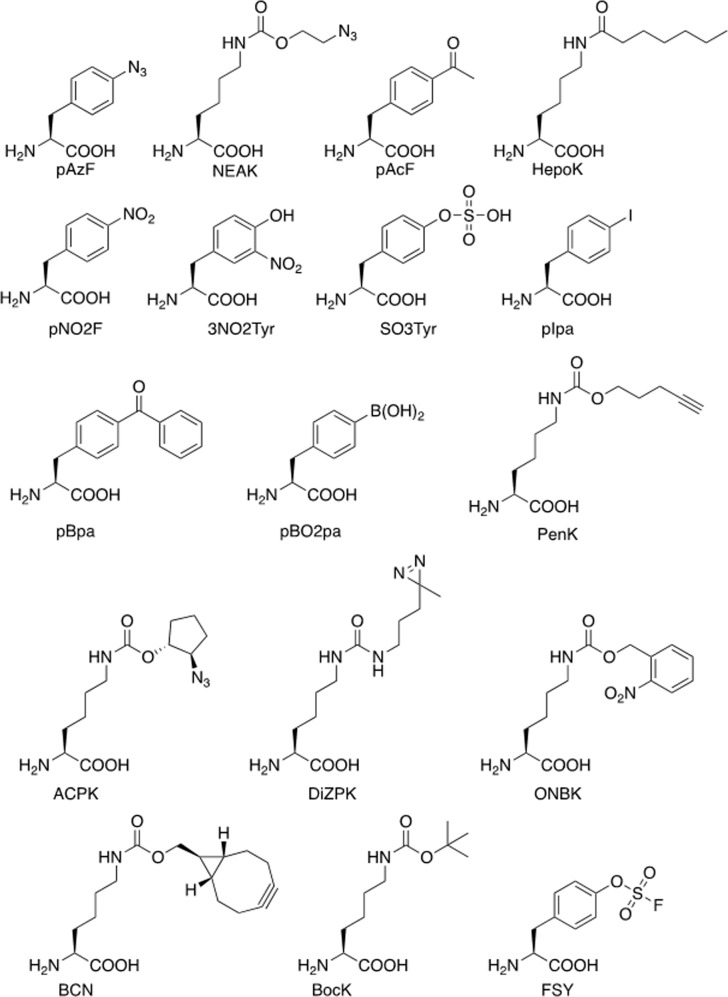
Non-canonical amino acids described in this review

Genetic code expansion has provided a valuable tool for protein PEGylation. One approach involves genetically encoding an ncAA (Fig. 10) containing a bioorthogonal chemical handle at the desired location into the target protein, followed by conjugation to PEG via a bioorthogonal reaction. In 2004, Deiters et al. reported the first ncAA-mediated mono-PEGylation method based on the genetic incorporation of p-azido-phenylalanine (pAzF) in yeast^249^. An alkyne-derivatized PEG chain was site-specifically attached to superoxide dismutase (SOD) via a copper(I)-catalyzed alkyne-azide cycloaddition (CuAAC) click reaction with pAzF, and the resultant SOD showed similar enzyme activity to the wild-type protein. Zhang et al.^250^ showed that this mono-PEGylation method could also be applied to interferon (IFN)-α2b. An azide-bearing ncAA, NEAK, was site-specifically incorporated into IFN-α2b at distinct locations, allowing orthogonal and stoichiometric conjugation to PEG via a copper-free cycloaddition reaction. Three resultant IFN-α2b variants showed significantly higher biological activities and better pharmacokinetic profiles than other variants and the wild-type molecule in rodent models. The above examples demonstrate that mono-PEGylation can be generally applicable to various proteins.

In 2011, Cho et al.^251^ reported the first clinical trial of recombinant proteins produced by genetic code expansion. Twenty human growth hormone (hGH) variants were made by incorporating *p-*acetylphenylalanine (pAcF) at distinct locations, followed by site-specific conjugation with PEG. One hGH variant with mono-PEGylation at residue 35, designated ARX201, presented favorable pharmacodynamic properties in GH-deficient rats, and comparable efficacy and safety to native hGH therapy, but with enhanced potency and reduced injection frequency, in adult GH-deficient patients. Wu et al.^252^ subsequently generated an hGH variant with combinatorial PEGylation at residues 35, 131, and 145 by genetically encoding multiple NEAK at these locations. The resultant multi-PEGylated hGH variant showed reduced immunogenicity and improved pharmacokinetic properties compared with mono-PEGylated hGH, without loss of bioactivity, and greater stability than mono-PEGylated hGH in rodent models. These examples illustrate the usefulness of genetic code expansion for optimizing therapeutic proteins and peptides.

An alternative site-specific PEGylation method involves directly introducing PEG-containing ncAAs into target proteins via genetic code expansion. Shozen et al. site-specifically incorporated ncAAs containing PEG4, PEG8, and PEG12 chains by suppressing a quadruplet codon using a cell-free translation system^253^. Tada et al. used a similar strategy to introduce longer PEG chains ranging from PEG4 to PEG24 into polypeptides by suppressing an amber stop codon^254^. Fu et al.^255^ recently introduced e-N-heptanoyl-l-lysine (HepoK) into GLP-1 (Fig. 10), and the resultant GLP-1 (HepoK) demonstrated stronger binding affinity towards human serum albumin (HSA) than wild-type GLP-1, as well as longer-lasting effects in terms of decreasing blood glucose levels, thus providing a powerful tool for studying protein lipidation.

Genetic code expansion and ncAAs have also been utilized to generate different types of vaccines, including peptide vaccines such as subunit, conjugated, and live-attenuated vaccines^256,257^. Grunewald et al.^258^ first demonstrated that incorporating an immunogenic ncAA into a protein of interest could break the immunological tolerance of self-proteins and evoke an immune response in animal models. Specifically, a single mutation of p-nitrophenylalanine (pNO2F) or phenylalanine was introduced into murine tumor necrosis factor-α (mTNF-α) at position 86 to generate mTNF-α (pNO2F) and mTNF-α (Phe), respectively. The resultant mTNF-α (pNO2F) induced a high-titer antibody response in mice, whereas mTNF-α (Phe) did not. In addition, the antibodies induced by mTNF-α (pNO2F) were found to be highly cross-reactive with native mTNF-α and protected mice against lipopolysaccharide (LPS)-induced death. In subsequent mechanistic studies, Grunewald et al.^258^ revealed that mTNF-α (pNO2F) mutants led to T cell-dependent polyclonal and anti-mTNF-α IgG antibody responses that were sustained for at least 40 weeks, and protected mice from severe endotoxemia induced by LPS challenge. This approach also elicited a high-titer IgG antibody response to murine retinol-binding protein, suggesting that this may be a generally applicable method for converting other weakly immunogenic self-proteins into vaccines. In a follow-up experiment^259^, besides ncAA (pNO2F), the incorporation of somatic mutations (Tyr in mTNF-α and Phe in EGF) and post-translational modifications (3NO2Tyr and SO3Tyr) at specific locations in self-proteins also elicited robust IgG antibody responses against the native proteins. The above results suggested that the site-specific incorporation of immunogenic ncAAs and certain natural post-translational modifications (PTMs) could break the immunological tolerance of self-proteins and produce therapeutic vaccines.

Wang et al.^260^ incorporated multiple ncAAs with a phenylalanine backbone into in *Mycobacterium smegmatis*, *Bacillus Calmette-Guérin*, and *Mycobacterium tuberculosis* to facilitate the study and development of tuberculosis vaccines. It is difficult to manipulate intact and live viruses using conventional chemical modification methods, due to the fragile nature and complicated assembly process of mammalian viruses. To overcome this challenge, Lin et al.^261^ reported the first example of site-specific incorporation of ncAAs into intact and live viruses followed by selective labelling, without loss of infectivity. Specifically, a panel of pyrrolysine analogues was genetically encoded into the envelope protein of hepatitis B virus (HBV) and assembled into live hepatitis D virus (HDV) in human hepatocytes, with stringent selectivity and high efficiency. By screening different incorporation sites, the viral infectivity was fully maintained. In addition, the ncAA-modified virus can be readily pulled down or conjugated via a copper(I)-catalyzed alkyne-azide cycloaddition click reaction. Wang et al.^230^ also applied an ncAA-mediated genetic switch to develop a live-attenuated HIV-1 vaccine. A panel of phenylalanine analogues was genetically encoded into the essential proteins of HIV-1 to control its replication, and HIV-1 replication could be precisely turned on and off via this approach. In a follow-up study, Yuan et al.^262^ merged the ncAA-mediated genetic switch into the viral genome and developed multi-cycle replicable HIV-1 based on amber suppression, representing a significant step towards the development of an HIV-1 vaccine. Chen et al.^263^ achieved precise control of HIV-1 replication via suppression of a quadruplet codon, which is not used by the native protein translation system, therefore minimizing the potential of proofreading and enhancing the safety of the vaccine. This method was also applied to influenza A virus^231^, and generated safe and effective live-attenuated vaccines that elicited robust protective immune responses in animal models, suggesting that ncAA-mediated live-attenuated vaccine is a generally applicable approach.

### Covalent peptide/protein drugs

Small molecule covalent drugs have many advantages compared with noncovalent drugs, such as increased biochemical efficiency and potency, improved pharmacokinetics, prolonged duration of action, reduced dosage and dosing frequency, and potent inhibition of intractable targets^264^. Safety concerns about their low selectivity and the potential immunogenicity of covalent drug-protein adducts mean that the development of small molecule covalent drugs has been intentionally avoided^265^. However, the development of activity-based protein profiling and other recent technologies mean that small molecule covalent drugs have regained attention, and several small molecule drugs that act by a covalent binding mechanism have been approved for marketing^266^.

Theoretically, covalent protein drugs should offer similar advantages to small molecule drugs. However, due to their inherent inability to form covalent bonds of natural proteins, the therapeutic potential of covalent protein drugs has not been fully explored. Li et al.^267^ recently reported on a proximity-enabled reactive therapeutics (PERx) strategy to develop covalent protein drugs. They genetically incorporated the latent bioactive ncAA, fluorosulfate-L-tyrosine (FSY)^268^, into human programmed cell death protein 1 (PD-1) at position 129 and showed that the resulting PD-1(FSY) formed covalent bonds selectively with its natural ligand, PD-L1, in vitro and in vivo. Strikingly, PD-1(FSY) significantly enhanced the bioactivities of human naïve T cells and engineered chimeric antigen receptor T cells, compared with wild-type PD-1. PD-1(FSY) showed more potent inhibition of tumor growth and had equivalent or greater anti-tumor effects than a therapeutic anti-PD-L1 antibody in several immune-humanized mouse models. They then applied PERx to the covalent inhibition of the HER2 receptor by a FSY-modified affibody, illustrating that PERx could provide a general platform for developing covalent protein drugs. Compared with noncovalent protein drugs, PERx drugs can be used in their original form and do not require additional modifications to extend their half-life, because the covalent binding decouples the drug efficacy from its pharmacokinetics. Moreover, PERx allows small-protein biologics such as PD-1 (15.6 kDa) to be used as therapeutics, thus greatly expanding the scope of therapeutic proteins. In addition, PERx can minimize the off-target effect due to the inherent affinity between the protein drug and its target, as well as the proximity-driven crosslinking mechanism of the latent bioactive ncAA. These advantages mean that the PERx strategy has the potential to provide a general platform to develop novel covalent protein drugs. The chemistry behind the PERx strategy and more examples of covalent proteins have been reviewed in detail elsewhere^269^.

Lipid and larger proteins are frequently conjugated to improve the pharmacokinetics of covalent peptide drugs^270–272^. Approved peptide drugs, such as liraglutide, semaglutide, and insulin degludec, were conjugated with C_14/16/18_ fatty acids, which increased their plasma circulation times and reduced their degradation during kidney elimination^270^. Two plasma proteins, serum albumin and immunoglobulin, are also used to prolong the peptide-circulation times by increasing their molecular weight, thereby exceeding the molecular weight cut-off for glomerular filtration. For example, this strategy was used to extend the half-life of dulaglutide and albiglutide, administered by once-weekly injections^273,274^.

### Developments in peptide drug delivery

Peptide modifications allow peptides to achieve better activity and plasma stability, and become more drug-like. However, the inherent properties of peptides mean that they are easily hydrolyzed by digestive enzymes in the stomach and intestine, and most peptide drugs are thus administrated by injection. Recent studies have investigated routes of peptide drug delivery to overcome these drawbacks^275^.

Co-formulation with permeation enhancers is a promising strategy to enable the oral administration of peptide drugs. Semaglutide conjugated with C_18_ fatty acid was approved for administration by once-weekly subcutaneous injection^276,277^, with greater plasma stability than other GLP-1 analogues. Even more encouragingly, the co-formulation of semaglutide with sodium N-[8-(2-hydroxybenzoyl amino]caprylate (SNAC) was approved for oral administration to treat T2DM. Co-formulation with SNAC prevents the destruction of semaglutide in the stomach by decreasing the efficacy of digestive enzymes. The hydrophobic SNAC molecules also increase the lipophilicity of semaglutide, thus improving its transcellular absorption through the gastric membrane and its transport into the systemic circulation^278,279^. Co-formulation with other permeation enhancers, enzyme inhibitors, and hydrogels have also been used to allow the oral administration of other peptide drugs, such as octreotide and insulin, which are now in clinical trials^280,281^. More strategies, including pulmonary administration, transdermal delivery, and the use of implantable pumps, are currently under investigation for the delivery of specific peptide drugs^282,283^, including the development of inhalable insulin and micro-implantable pumps for insulin delivery. We expect these technologies to be applied for more peptide drugs in the coming years.

## Current development and application of therapeutic peptides in diseases

### Therapeutic peptides in the treatment of diabetes mellitus

**Fig. 11:**
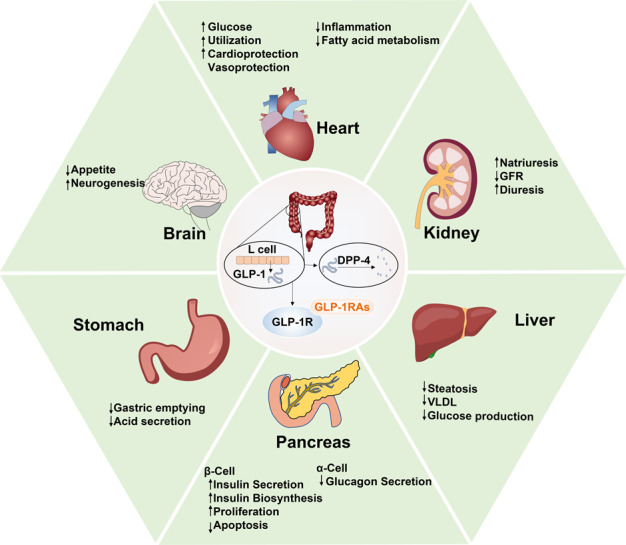
Mechanisms of GLP-1 and GLP-1RA peptide drugs in regulation of T2DM. GLP-1 and GLP-1RA peptide drugs treat T2DM by regulating multiple organs functions, such as reducing gastric emptying and gastric acid secretion, reducing appetite, promoting cardiac glucose utilization, accelerating renal natriuresis and diuresis, minimizing glucose production in the liver and increasing insulin secretion in the pancreas

T2DM is caused by an acquired insulin deficiency and is common in middle-aged and older people. T2DM has been successfully treated with peptide drugs, including GLP-1 receptor agonists (GLP-1RAs) and the best-known peptide drug, insulin. GLP-1 is an endogenous growth hormone secreted by L-cells in the ileum. Its receptors are present in pancreatic β-cells, the peripheral and central nervous systems, heart and blood vessels, kidneys, lungs, and gastrointestinal mucosa (Fig. 11). GLP-1 interacts with its receptor to stimulate islet β-cells to secrete insulin, inhibit the release of glucagon by islet α-cells, increase satiety, and delay gastric emptying in a glucose-dependent manner^284^. Endogenous GLP-1 is degraded by dipeptidyl peptidase-4 (DPP-4) and is rapidly inactivated. In order to prolong the stimulation time of GLP-1 receptors, synthetic GLP-1RAs are required to prevent its degradation. Since the first GLP-1RA, exenatide, was approved by the US Food and Drug Administration (FDA) in 2005, several GLP-1RAs have entered the clinic, including liraglutide (2009), lixisenatide (2013), dulaglutide (2014), and semaglutide (2017)^285^. After injection, these GLP-1RAs effectively reduce glycosylated hemoglobin and average blood glucose levels and improve fasting blood glucose^286^.

Some GLP-1RAs are also effective or the treatment of some complications of T2DM. Diabetic nephropathy is one of the most dangerous complications of T2DM, leading to severe effects on kidney function in diabetic patients, with clinical manifestations including proteinuria and decreased glomerular filtration rate (GFR). In a study of 35 patients with T2DM, lixisenatide reduced the absolute and partial excretion of magnesium, calcium, and phosphate by inhibiting the proximal tubule sodium-hydrogen antiporter 3 (NHE3) and thus increasing the absolute and partial excretion of sodium, chlorine, and potassium and increasing urine pH values compared with insulin glargine^287^. In addition, in a study of 30 T2DM patients, liraglutide significantly reduced GFR, urinary albumin excretion rate, and partial albumin excretion^288^. GLP-1RAs can reduce GFR by increasing sodium efflux to the macula densa, increasing tubulo-glomerular feedback and vasoconstriction of afferent arterioles, and may also reduce albuminuria by reducing plasma renin activity, reducing renal oxidative stress, and increasing natriuresis^289^. However, the extent to which these effects are mediated by GLP-1R remains to be determined. Recent studies have confirmed that the metabolites of GLP-1 retain important antioxidant and anti-apoptotic activities, which are independent of GLP-1R^290^. Cardiovascular disease remains the leading cause of death in patients with T2DM, and the prevention and treatment of cardiovascular complications should thus be considered when choosing treatments for T2DM. GLP-1RAs have been shown to play a beneficial role in cardiovascular disease. Recent clinical trials found that only liraglutide and semaglutide had advantages in terms of cardiovascular benefits, although the mechanism is still unclear and may be anti-atherosclerotic^48^. The protective effects of other GLP-1RAs on cardiovascular disease are not obvious, but they have no harmful effects on other safety parameters, and the risk-benefit distribution of GLP-1RAs is thus well-balanced^291^. GLP-1RAs also showed therapeutic effects on obesity symptoms in patients with T2DM. Thondam reported that a patient with severe hypothalamic obesity and various obesity-related complications, including T2DM, responded well to exenatide, with significantly improvements in weight and blood glucose control, possibly through a central regulatory mechanism increasing satiety and reducing energy intake^292^. A study of 25 obese patients with T2DM showed that patients treated with metformin and sulfonylurea/DPP-4 inhibitors for 6 months who took GLP-1RA (exenatide19, six cases) had significantly reduced average body weight, glycosylated hemoglobin, and intrahepatic lipids^293^. Body mass index and fat thickness also decreased significantly in 25 patients with T2DM treated with exenatide and liraglutide for 3 months^294^. T2DM can lead to bone brittleness and increase the risk of bone-related complications such as fractures and poor fracture healing. Experimental studies found that GLP-1RAs had a significant positive effect on bone quality and strength, possibly by improving the blood supply to the bone necessary for bone health^295^. In one study, liraglutide was applied to ovariectomized rats with T2DM, followed by high-throughput sequencing of bone marrow-derived exosome micro RNAs (miRNAs). Liraglutide was shown to cause significant changes in exosome miRNAs targeting the insulin signaling pathway, and changes in the Wnt/β-catenin signaling pathway mediated by bone marrow exosomes were implicated in the osteoprotective effect^296^.

The most common side effects of GLP-1RA treatment are gastrointestinal-related adverse reactions (i.e., nausea, vomiting, and diarrhea) and injection-site reactions, while long-acting GLP-1RAs have fewer side effects, a lower administration frequency. and better compliance. Metformin is still the first-line drug for the treatment of T2DM in the clinic. According to the European Diabetes Research Association and the American Diabetes Association, GLP-1RAs, sulfonylureas, thiazolidinediones, DPP-4 inhibitors, sodium-glucose cotransporter 2 inhibitors, and insulin are recommended as complementary drugs for patients whose blood sugar is not sufficiently controlled by metformin alone^297^. However, based on the many other benefits of GLP-1RAs in addition to blood glucose control, including renal protection, reduced risk of cardiovascular disease, weight control, no risk of hypoglycemia, benefits for skeletal symptoms, and low-frequency side effects, GLP-1RAs will play an essential role in the treatment of T2DM in the future.

### Therapeutic peptides in the treatment of cardiovascular disease

Among non-communicable diseases, cardiovascular disease is now the leading cause of death and morbidity worldwide^298^. Hypertension is one of the main risk factors for the development of cardiovascular disease, and is considered to be caused by high activity of the renin-angiotensin-aldosterone system (RAAS) and sympathetic nervous system,as well as sodium retention^299^. The function of angiotensin-converting enzyme (ACE) in the RAAS is to cleave angiotensin I into angiotensin II, to contract blood vessels and indirectly increase blood pressure, while ACE2 hydrolyzes angiotensin II into vasodilator angiotensin (1-7) to indirectly reduce blood pressure^300^. Targeting the RAAS thus represents an ideal strategy for controlling cardiovascular diseases. Synthetic angiotensin II was approved by the FDA in 2017 for increasing blood pressure via intravenous infusion in adults with septicemia or other distributed shock^301^. Four peptides (WPRGYFL, GPDRPKFLGPF, WYGPDRPKFL, and SDWDRF) isolated and screened from *Tetradesmus obliquus* microalgae by Montone et al. showed inhibitory activity against ACE^302^. Liao et al. found that the tripeptide IRW, derived from egg white, reduced blood pressure in spontaneously hypertensive rats by up-regulating the expression of ACE2. These studies indicate the potential application of food-derived peptides targeting RAAS for the treatment of cardiovascular diseases^303^.

**Fig. 12:**
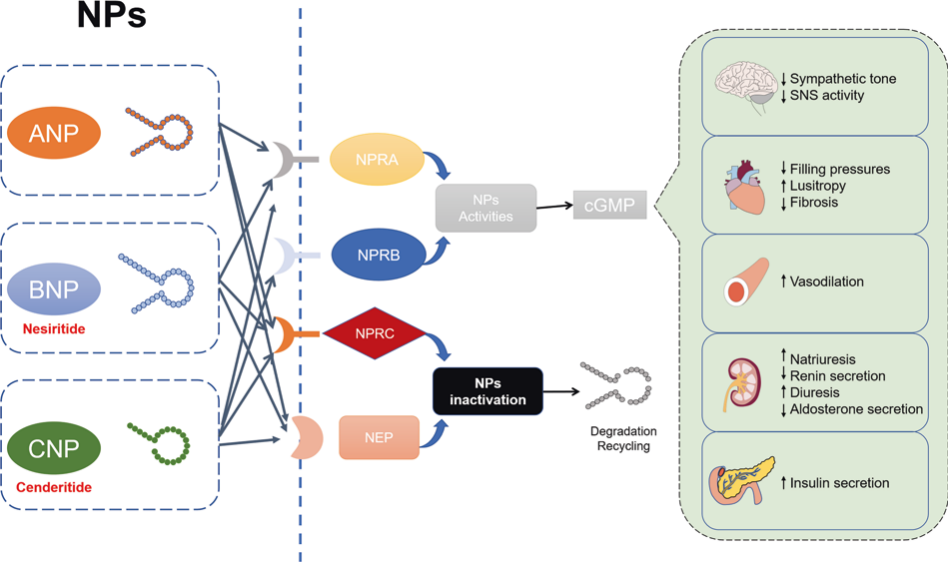
Mechanism of natriuretic peptide (NPs) regulation. Atrial natriuretic peptide (ANP), brain natriuretic peptide (BNP), and C-type natriuretic peptide (CNP) regulate cardiac and vascular homeostasis through binding to their receptors (NPR-A, -B and -C) to reduce sympathetic tone, fibrosis and renin secretion to treat cardiovascular diseases

Natriuretic peptide (NPs)^304,305^, including atrial natriuretic peptide (ANP), brain natriuretic peptide (BNP), and C-type natriuretic peptide (CNP), are essential regulators of cardiac and vascular homeostasis (Fig. 12). Targeting NPs is thus another practical strategy for the prevention or treatment of cardiovascular diseases. Nesiritide is a recombinant human BNP that was approved by the FDA in 2001 for the treatment of acutely decompensated heart failure in patients with resting or mild dyspnea^306^; however, it has not been widely used due to its low specificity and limited safety^307^. NPs act mainly through NPR-A and/or NPR-B receptors, while NPR-C is mainly used for scavenging NPs^308^. Cenderitide is a dual NPR-A/NPR-B agonist composed of CNP and the C-terminal of dendroaspis natriuretic peptide isolated from the green mamba snake^309^. Cenderitide is currently in clinical research and has shown safety and potential for the treatment of heart failure and renal failure^309,310^. In addition, some peptides that are beneficial to cardiovascular disease are being tested in animals. For example, infusion of vasoactive intestinal peptide increased the concentration of myocardial vasoactive intestinal peptide and reversed existing myocardial fibrosis in rats^311^, and cyclopeptide RD808 neutralized the β_1_-adrenergic receptor, thus attenuating myocardial injury induced by the β_1_-adrenergic receptor in mice^312^. The central adrenocorticotropin-releasing factor (CRF)-related peptide system is currently attracting increasing attention as a target for the prevention of cardiovascular disease^313^. There is a complex relationship between the CRF-related peptide system and the cardiovascular system, but its exact regulatory role in cardiovascular function remains to be determined. In addition, the activity of circulating DPP-4 was increased and flow-mediated dilation was decreased in patients with T2DM. Flow-mediated dilation is a recognized alternative marker of endothelial dysfunction and a predictor of future cardiovascular events, suggesting that DPP-4 may be a potential target for preventing cardiovascular disease^314^.

### Therapeutic peptides in the treatment of gastrointestinal diseases

#### Therapeutic peptides in the treatment of intestinal disease

In the human body, gastrointestinal flora constitutes a complex micro-ecosystem. Typically, the gastrointestinal flora in the human body constitutes a complex micro-ecosystem. Typically, the epithelium regulates the composition of the intestinal flora at the intestinal mucosal interface by providing a physical barrier and secreting various antimicrobial factors, including antimicrobial peptides (AMPs). The dominant flora (physiological flora) and weak flora (pathogenic bacteria) maintain a dynamic balance^315^, which is disrupted in various intestinal diseases caused by exotic bacteria, viruses, and parasites, food poisoning, adverse drug reactions, and genetic factors, such as enteritis, constipation, intestinal ulcers, and inflammatory bowel disease (IBD). The extensive use of antibiotics may further reduce the biodiversity of symbiotic bacteria, which is not conducive to treatment and may even aggravate the disease; for example, individuals affected by IBD are more likely to have used antibiotics within 2-5 years before diagnosis^316^. Peptide drugs have attracted much attention in this field because of their specificity, efficacy, and low toxicity.

Significant changes in the normal intestinal flora and the destruction of host-microbial symbiosis may be the key to the development of IBD^317^. IBD, including Crohn’s disease and ulcerative colitis, is caused by an intestinal immune response, and the associated inflammation is caused by the interaction between environmental and genetic factors^318^. However, the specific pathogenesis of IBD is still unclear and there is currently no effective cure. Intestinal microbial diversity is significantly reduced in patients with IBD^319^, and the two dominant phyla Firmicutes (Lachnospiraceae) and Bacteroidetes, were significantly decreased while the phylum Proteus was significantly increased^320^. Substantial evidence has indicated a key role for members of the phylum Proteus in IBD^321^. Proline-arginine-39, a small cationic AMP that is naturally secreted by porcine bone marrow and lymphoid tissue, has demonstrated antibacterial, immunomodulatory, and intestinal epithelial repair functions and may provide a safe alternative therapy for IBD^322^.

**Fig. 13:**
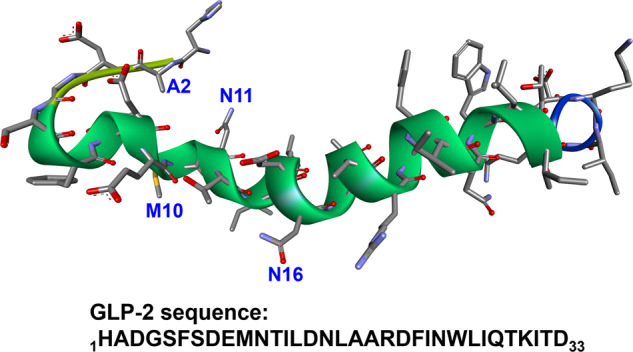
The structure and sequence of GLP-2 (PDB: 2L63)^325^

Patients with Crohn’s disease are often treated by bowel resection^323^, leading to short bowel syndrome (SBS). Damage to the small intestine and abnormal shortness of the small intestine at birth may also cause SBS, which is defined as symptoms associated with a persistent length of the residual small intestine of <200 cm^324^. GLP-2 is produced by intestinal endocrine L cells and various neurons in the central nervous system (Fig. 13)^325^ and has recently received extensive attention for the treatment of SBS. GLP-2 has demonstrated various beneficial effects, including stimulating crypt cell growth, reducing intestinal cell apoptosis, promoting intestinal mucosal dilatation, inhibiting gastric acid secretion and gastric emptying, stimulating intestinal blood flow, strengthening intestinal barrier function, reducing anti-inflammatory injury, and promoting nutrition and liquid absorption^326–328^. GLP-2 also regulated the expression of amino acid transporters and directly activated mTORC1 to increase the absorption of amino acids in the intestinal epithelium^326^. Some specific amino acids (including glutamine, glutamate, arginine, glycine, lysine, methionine, and sulfur-containing amino acids) have also been shown to play an important role in maintaining intestinal integrity, including preventing intestinal atrophy, improving intestinal barrier function, and reducing inflammation and apoptosis^329^. Endogenous GLP-2 is easily degraded by DPP-4; however, the GLP-2 analogue teduglutide prolongs the half-life from 7 minutes to about 2-3 hours by substitution of alanine by glycine in the second position of the N-terminal of GLP-2, effectively preventing its degradation by DPP-4^330^. Clinical studies have shown that teduglutide can effectively reduce or eliminate the need for parenteral nutrition and/or intravenous infusion support^331^, while the application of teduglutide in young pigs with distal ileectomy significantly increased the weight per unit weight and protein synthesis of the remnant intestine^332^. Teduglutide was approved by the FDA for clinical use in SBS patients in 2012. Wiśniewski et al. designed a series of GLP-2 analogues, including 2-glycine substitution, 10-norleucine substitution, 11- and/or 16-hydrophobic substitution, many of which were more effective against GLP-2R than natural hormones, showing good receptor selectivity and low systematic clearance. Among these, the peptide ([_2_Gly, _10_Nle, _11_DPhe, _16_Leu] hGLP-2-(1−33)-NH_2_) was selected as a candidate for clinical development^333^. GLP-1 from the proglucagon family has similar functions to GLP-2 and has been suggested for the treatment of SBS. In one study, five patients with SBS showed improved stool frequency and morphology after treatment with the GLP-1 agonist exenatide^334^. Similarly, GLP-1 reduced diarrhea in nine SBS patients, but was less effective than GLP-2, while the combination of GLP-1 and GLP-2 was superior to administration of either alone^335^. Glicentin, another member of the proglucagon family, also appears to be involved in many processes such as enteral nutrition, exercise, and gastric acid secretion, indicating the prospect of developing glicentin-like peptides^336^. Other growth factors such as EGF, erythropoietin, and hepatocyte growth factor have also shown therapeutic potential in SBS. The combination of EGF and GLP-2 increased the length of the small intestine in three newborn piglet models of SBS, indicating that EGF has therapeutic potential in neonatal SBS^337^. Erythropoietin protected intestinal barrier function and protected the gastrointestinal tract from ischemia/reperfusion injury by stimulating the expression of tight junction proteins in animal models^338^, and enteral injection of hepatocyte growth factor reduced the incidence and severity of necrotizing enterocolitis in rats^339^.

*Clostridium difficile* toxin A produced by pathogenic strains of *Clostridium difficile* causes diarrhea and inflammation and even severe pseudomembranous colitis in infected people^340^. Periplanetasin-2 (YPCKLNLKLGKVPFH) is an AMP isolated from the American cockroach by Ji et al., which blocks the mucosal damage and inflammation induced by *Clostridium difficile* toxin A^341^, and was recently identified as a candidate drug for relieving/treating pseudomembranous colitis caused by *Clostridium difficile* toxin A^341,342^. The 9-mer disulfide dimer peptide CopA3 (LLCIALRKK) isolated from the Korean dung beetle significantly improved the small intestinal inflammatory response (acute enteritis) induced by *Clostridium difficile* toxin A and completely blocked the inflammatory response and subsequent fatal response of chronic colitis induced by sodium dextran sulfate in mice^343^. Food poisoning caused by *Clostridium perfringens* type A is related to several important human gastrointestinal diseases, and is thought to be mediated by the production of *Clostridium perfringens* enterotoxin (CPE) combined with human intestinal claudins. Archana et al. found that preincubation or co-incubation of CPE with the claudin-4 extracellular loop ECL-2 peptide significantly inhibited CPE-induced luminal fluid accumulation and histological lesions in rabbit intestinal loop^344^, suggesting that the synthetic peptide ECL-2 may represent a potential strategy for preventing intestinal histological damage caused by *Clostridium perfringens* type A. Cathelicidin secreted by human colonic epithelium is another AMP with a wide range of antimicrobial and immunomodulatory functions. Recent studies have shown that human cathelicidin helped early colonic epithelial cells defend against enterogenous *Salmonella typhimurium* by preventing bacterial invasion and maintaining the integrity of the epithelial barrier, possibly by producing Toll-like receptor-4 and pro-inflammatory cytokines^345^. In addition, enterovirus infection has also been shown to stimulate the expression of AMPs. Chen et al. found that small ribonucleic acid virus infection increased the expression and secretion of human β defensin-3 in intestinal epithelial cells, and human β defensin-3 had extracellular anti-enterovirus activity^346^.

Patients with cystic fibrosis (CF) usually also have intestinal obstruction and constipation, which may develop into distal intestinal obstruction syndrome^347^. The guanylate cyclase C (GCC) receptor agonist, linaclotide, was approved by the FDA in 2012 for the treatment of chronic constipation. Linaclotide has also been shown to improve intestinal transport in CF model mice, although further studies are required to evaluate its effects on the intestinal pathology in CF patients. The NHE3 inhibitor tenapanor improved gastrointestinal transport in CF mice by targeting inhibition of sodium absorption^348^, indicating that inhibition of GCC signal transduction and NHE3 may be a suitable target for the treatment of constipation in patients with CF.

In addition to drug-derived peptides, peptides may also be food-derived. Asn-Pro-Trp-Asp-Gln (NPWDQ), a peptide obtained by hydrolyzing casein (a major milk protein), significantly inhibited the penetration of the food allergen, ovalbumin, into human intestinal Caco-2 cells, suggesting that this peptide might improve the function of the intestinal epithelial barrier^349^. β-Casofensin is a peptide found in fermented milk, and in vivo experiments found that early administration of β-casofensin reduced indomethacin-induced intestinal injury and inflammation by protecting goblet cells and promoting wound healing^350^. Indomethacin-induced intestinal damage has the same clinical, histological, and pathophysiological characteristics as Crohn’s disease^351^, suggesting that β-casofensin may be a potential adjuvant therapy for Crohn’s disease.

Peptide drugs also have broad prospects in the treatment of intestinal diseases in livestock. Liu et al. developed a modified synthetic peptide KR-32 using natural snake AMP as the raw material. KR-32 improved the malabsorption of fatty acids, total digestibility of ether extract, and intestinal morphology in piglets treated with enterotoxigenic *Escherichia coli* K88, indicating the potential medicinal value of KR-32^352^. C-BF is a peptide derived from cathelicidins, as the most prominent AMP family, and is considered to be the most promising substitute for antibiotics. C-BF significantly improved the growth of weaned piglets and improved the structural and developmental damage to the small intestine caused by LPS, indicating that C-BF may be a potential treatment for intestinal damage caused by LPS/pathogens^353^.

#### Therapeutic peptides in the treatment of gastric disease

The gastric mucosa is one of the most vulnerable tissues in humans and animals, and gastric diseases are a common problem. *Helicobacter pylori* infection, non-steroidal anti-inflammatory drugs, alcohol, smoking, mood, and stress are the main factors responsible for stomach damage, which in turn leads to gastritis and ulcers. Stomach disease can develop into a chronic disease in the absence of timely treatment or with improper treatment, and sustained long-term damage greatly increases the risk of gastric cancer. Gastric cancer is currently the fourth most frequently diagnosed cancer worldwide, and the third and fifth leading causes of cancer-related deaths among men and women, respectively.

Although no peptides have yet been approved for the treatment of gastric diseases, the roles of peptides in gastric diseases, including endogenous and exogenous peptides, have been widely studied in the past decade. Calcitonin gene-related peptide (CGRP) is widely distributed in the gastrointestinal system and is the primary neurotransmitter of capsaicin-sensitive sensory nerves. These sensory nerves are involved in protecting the gastric mucosa from various stimuli, and CGRP acts as potential mediator in this process, increasing gastric mucosal blood flow, inhibiting gastric acid secretion, and preventing apoptosis and oxidative damage^354^. In addition to CGRP, the nitric oxide synthase-nitric oxide (NOS-NO) and cyclooxygenase-prostaglandin (COX-PG) systems have similar protective effects on the stomach^355^. CGRP, NO, and PG are considered to be the terminal mediators of gastric protection, and to mediate the gastroprotective effects of many endogenous peptides^356^. The primary pathogenesis of ethanol-induced gastric injury is gastric microvascular injury. As a peptide derived from the nerve growth factor inducible (VGF) gene, TLQP-21 mediated by constitutive NO, PGE2, and somatostatin, showed that central rather than peripheral injection could attenuate ethanol-induced gastric injury in a dose-dependent manner^357,358^. Novokinin (Arg-Pro-Leu-Lys-Pro-Trp) is an effective vasodilator and antihypertensive peptide modified by ovokinin, with high selective affinity for angiotensin II type 2 (AT2) receptors. Zhang et al. found that novokinin inhibited basal gastric acid secretion after intracerebroventricular in a dose-dependent manner and protected the gastric mucosa from alcohol-induced injury, by mediating the AT2 receptor-PG pathway^359^. These results indicated the value of TLQP-21 and novokinin for the treatment of gastric injury. A peptide extract obtained from the hydrolysis of waste beer yeast protein (especially < 3 kDa) reduced gastric mucosal injury in rats, indicating the potential value of yeast peptide extract for the treatment of gastric diseases^360^.

Animal stress-induced gastric injury is often used as a model to study the mechanism of stress-induced stomach diseases. The AMP hepcidin is thought to be produced by parietal cells regulating gastric acid production, and acid secretion was significantly decreased in hepcidin-knockout mice, suggesting that hepcidin may be related to the occurrence of gastric ulcers under stress conditions^361^. Nesfatin-1 belongs to the anorexia peptide family, which exists in neurons and endocrine cells of the intestinal tract. Studies by Alexandra et al. showed that nesfatin-1 had a significant protective effect on the stomach in rats exposed to water immersion restraint stress. The mechanism was related to decreased gastric juice secretion, hyperemia mediated by the COX-PG and NOS-NO systems, and activation of the vagus nerve, sensory nerve, and vanillin receptor^355^. Chronic mild stress can cause gastric ulcers in rats, and the somatostatin analogue octreotide can alleviate gastric ulcers by inhibiting apoptosis, inflammation, and oxidation^362^. Central rather than peripheral injection of oxytocin can eliminate the enhanced postprandial gastric contraction induced by restraint stress in rats, thus reducing delayed gastric emptying, suggesting that oxytocin may be a candidate drug for the treatment of stress-related gastrointestinal motility disorders^363^.

Gastric cancer is a severe stomach disease. Several peptides have shown therapeutic prospects in gastric cancer. GEBP11 is a new nine-amino acid homing peptide screened and identified by phage-display technology. GEBP11 selectively binds to human umbilical vein endothelial cells and tumor vessels, suggesting that it may be an important candidate for tumor imaging and targeted drug delivery^364^. Treatment with the iodine 131-labeled bifid PEGylated GEBP11 trimer (^131^Imur2PEG-(GEBP11)_3_) significantly inhibited the growth of human gastric cancer xenografts in nude mice and prolonged the survival time, indicating that ^131^Imur2PEG-(GEBP11)_3_ may be a suitable candidate for peptide-targeted therapy of gastric cancer and a drug carrier for antiangiogenic therapy of gastric cancer^365^. *Helicobacter pylori* infection is one of the most important causes of gastric cancer. H-P-6 (Pro-Gln-Pro-Lys-Val-Leu-Asp-Ser), an active peptide isolated from microbial hydrolysate of *Chlamydomonas sp*., has been shown to resist *Helicobacter pylori*-induced carcinogenicity. H-P-6 down-regulated phosphoinositide 3-kinase/Akt signal transduction and β-catenin nuclear translocation by inhibiting EGFR activation, and effectively inhibited *Helicobacter pylori*-induced human gastric adenocarcinoma cell (AGS) proliferation and migration without inhibiting bacterial viability or AGS cell invasion^366^. Zhang et al. synthesized the AMP pexiganan and its nanoparticles (PNPs), which demonstrated anti-*Helicobacter pylori* activity and stronger scavenging ability against *Helicobacter pylori* in mouse stomach than pexiganan, and showed potential for the treatment and prevention of *Helicobacter pylori*-related gastric diseases^367^. TFF1 is a mucin-related gastric mucosal cell secretory peptide. The expression of TFF1 was up-regulated in the gastric antrum in the acute phase rather than the chronic phase of *Helicobacter pylori* infection in mice, and was negatively correlated with the inflammatory response, indicating that TFF1 may help cells resist the development of bacteria and chronic inflammation^368^. TFF2, a member of the same family, has been shown to interact with gastrin MUC6 to stabilize the gastric mucus barrier and maintain gastric mucosal integrity^369^.

Peptides have also been shown to play a regulatory role in terms of gastric motility. Peripheral injection of GLP-2 increased gastrointestinal blood flow and gastric mucosal blood flow by increasing CGRP and endogenous PGs rather than NO^370^. Exogenous GLP-1 caused the release of NO into the gastric antrum through nerves in an isolated whole-stomach model, thus reducing gastric motility in mice^371^. However, whether the approved GLP-1/2 derived peptides have similar effects still needs to be investigated. BNP has the property of dilating blood vessels and can increase visceral perfusion and oxygenation, and recombinant BNP has been shown to increase hemoglobin oxygenation in the gastric mucosa microvasculature^372^. Motilin and ghrelin belong to the same peptide family, and these hormones play an important role in the regulation of gastrointestinal motility. Ghrelin and motilin can synergistically stimulate strong gastric contraction in vitro and in vivo^373^. Motilin and the combination of motilin and ghrelin stimulated gastric acid secretion in the shrew *Suncus* through the histamine-mediated pathway^374^.

### Therapeutic peptides in the treatment of cancer

Traditional cancer treatments include surgery and radiotherapy, which have limited effects in patients with advanced cancer. The subsequent development of targeted therapy and immunotherapy have significantly improved the survival rate of cancer patients. Targeted therapy takes advantage of the reliance of tumor cells on specific molecules or signaling pathways to kill tumor cells using a “guided missile” approach^375^. Immunotherapy drugs do not attack tumor cells directly but modulate the patient’s own immune system and attack tumor cells by targeting immune checkpoints^376^. PD-1/PD-L1 is a well-known immune checkpoint, and five monoclonal antibodies against PD-1/PD-L1 interaction have been approved by the FDA for cancer treatment. However, antibodies have disadvantages including high cost, poor oral suitability, and high immunogenicity. Peptides have also attracted attention in the field of tumor diagnosis and treatment because of their small size, high affinity, easy modification, and low immunogenicity. Some modified peptides have also demonstrated good stability. For example, Carvajal et al. developed stable α-helical peptides as inhibitors of MDM2 and MDMX for the treatment of p53-dependent cancer^377–379^.

The short half-life of natural peptides in vivo means that peptides targeting various abnormally expressed receptors in tumor cells are usually modified peptide analogues. There are three main methods for the production of these peptides, each with its own strengths and weaknesses: 1) derivation from natural proteins; 2) chemical synthesis and reasonable engineering based on structure; and 3) screening of peptide libraries^380^. Among these, phage-display technology is a traditional and widely used method, with the advantages of simple operation and effective screening of a large number of different peptides^381^.

**Fig. 14:**
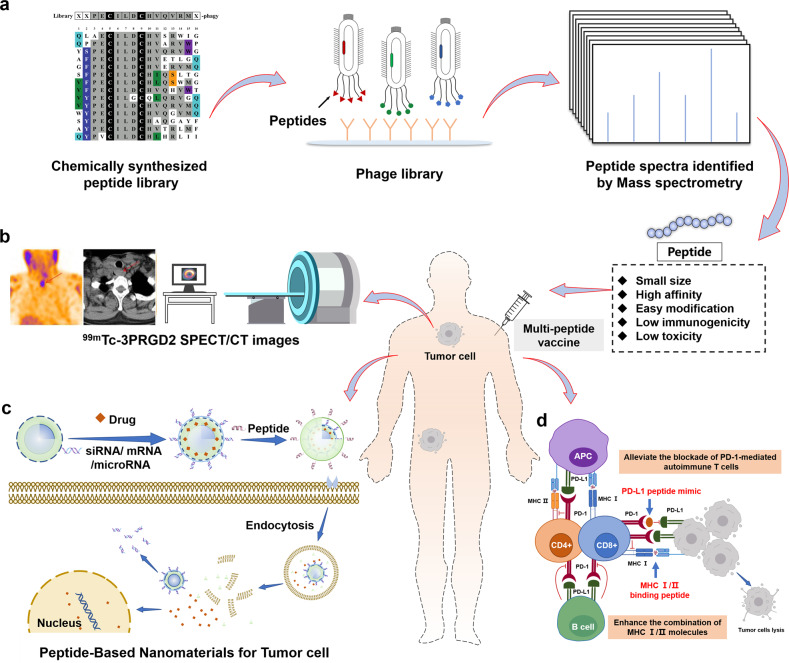
Application of peptides in tumor therapy. **a** Screening and identification of peptide candidates from chemically synthesized peptide library and phage library. **b** Using radiolabeled, dye-labeled, or other designed peptides as probes for tumor diagnosis and imaging. **c** Application of peptide-conjugated nanomaterials in tumor therapy. **d** Using peptide vaccine and targeting peptides in tumor immunotherapy and targeted therapy

Peptides can be applied in tumor therapy in four main ways: 1) using radioisotopes, dyes, or other reported molecular-labeled peptides as probes for tumor diagnosis and imaging; 2) using peptide-coupled nanomaterials for tumor therapy; 3) using peptide vaccines to activate the immune system for prevention; and 4) using peptides alone as targeted drugs (Fig. 14)^382–384^.

Peptide-based imaging probes bind to receptors specifically expressed in the tumor. These receptors can either be expressed on the cell surface, such as αvβ3 integrin (RGD peptide), EGF receptor, somatostatin receptor, neurotensin receptor, and transferrin receptor; intracellularly, such as Bcr/Abl, cyclin A, and cyclin kinase; or in extracellular matrices, such as fibronectin, matrix metalloproteinases, and prostate-specific antigen^382^. The locations of probes can be visualized by single-photon emission computed tomography/computed tomography scanning to indicate the tumor distribution. This technique has been applied for early tumor diagnosis and surgical resection. Several probes have been developed, such as octreoscan and depreotide, as radiolabeled conjugates of somatostatin-like peptides, have been approved by the FDA for imaging of tumors, such as neuroendocrine tumors and lung cancer^385,386^. Unfortunately, depreotide has been withdrawn. Radiolabeled peptides based on RGD peptides have recently received attention, and a series of probes have been synthesized, including (99m) Tc-3PRGD2, which can be used to detect differentiated thyroid cancer by negative whole-body scan of radioactive iodine^387^. Lutetium 177 dotatate is a radiolabeled somatostatin analog recently approved for the treatment of somatostatin receptor-positive gastroenteropancreatic neuroendocrine tumors^388^. It binds to somatostatin receptors and then releases radioactive Lutetium 177 into the tumor cells, which induces cellular damage via the formation of intracellular free radicals^389,390^.

Based on the same principle as peptide-based probes, internal radiotherapy can be achieved by labeling β-emitters on peptides; however, this is notably limited by radiation damage to normal target tissues with positive receptors, either near or far from the tumor^384^. A possible alternative to reduce the potential side effects involves delivering the peptides to tumors coupled to anti-cancer drugs, genes, and RNAs (small interfering RNA ([siRNA]/miRNA/mRNA)^391^. AN-152 and AN-207 are luteinizing hormone-releasing hormone analogues coupled to adriamycin, with anticancer activity against luteinizing hormone-releasing hormone receptor-positive cancers. The results of phase I and II clinical studies showed that the drug was effective for the treatment of breast cancer, endometrial cancer, and ovarian cancer, with moderate toxicity and side effects^392^. Chen et al. designed PEGylated liposome-polycation-DNA (LPD) nanoparticles and obtained LPD-PEG-NGR by modifying the NGR peptide targeting tumor-specific receptor aminopeptidase N. LPD-PEG-NGR delivered siRNA into solid tumors in mice with systemic, specific, and effective delivery, and by delivering c-myc siRNA, it effectively triggered the apoptosis of tumor cells by down-regulating the expression of c-myc, thus inhibiting the growth of some tumors^393^. In addition, tumor-penetrating peptides screened by phage screening in vivo can effectively deliver covalently coupled and co-administered drugs to the depths of tumor tissues^394^. These results indicate that peptide-based drug delivery systems have important potential for the treatment of tumors.

Antigenic peptides from specific target proteins can act as anticancer peptide vaccines by binding to the major histocompatibility complex (MHC) on antigen-presenting cells, to trigger the anti-tumor effects of helper or cytotoxic T cells. EGFRs, such as EGFR1 and HER2, are well-known targets for cancer treatment. The peptide vaccine TERT572Y, based on the HER2 structure, was used in 46 patients with advanced non-small-cell lung cancer. Subcutaneous injection of TERT572Y induced a TERT-specific immune response and significantly prolonged survival^395^. Manijeh et al. calculated and predicted potential epitopes by PEPOP and selected various peptide sequences from the extracellular domain of HER2 as candidate sequences. They then evaluated the binding affinity of these candidate peptides to MHC I and II molecules by molecular docking, to find the most stable binding structure between peptides and MHC I and II molecules, and selected MHC class I- and II-binding peptides as breast cancer peptide vaccines^396^. However, most clinical trials of peptide vaccines have failed to demonstrate excellent therapeutic effects, and peptide vaccines have thus received little attention. Nevertheless, Takumi et al. argued that the main reason for the lack of success in clinical studies of most cancer vaccines, including peptides, was due to their poor immunogenicity, and suggested that optimization of peptide formulations, adjuvants, and administration routes would achieve ideal results^397^. Peptides, known as cell-penetrating peptides (CPPs), can also be used as drug carriers to transport other peptides, proteins, DNAs, small RNAs, and drugs into cells^398^. The CPP-drug construct comprising nerinetide with the CPP Tat was used to deliver nerinetide across the blood–brain barrier and into neurons^399^. Peptides have also shown a promising delivery function by coupling to antigens to induce antigen-specific immune tolerance and reduce the risk of off-target responses^400^. Tsoras used peptide nanoclusters to improve peptide subunit vaccine immunogenicity for oncofetal antigen^401^.

In addition to being used as drug carriers and vaccines, peptides can also exert anti-tumor effects by binding to target receptors. Among these, the most popular peptides are those targeting the PD-1/PD-L1 signal pathway. Boohaker et al. designed a PD-L1 peptide mimic, PL120131, which can interfere with the interaction of PD-1/PD-L1 by binding to PD-1. PL120131 maintained the survival and activity of co-cultured T cells better than PD-1 antibody in a 3D co-culture model^402^. Based on peptides binding to PD-1 and PD-L1, Zhou et al. designed the self-inhibitory peptides DS-I and DS-II and their cyclic peptide forms, which showed strong affinity to PD-1^403^. Abbas et al. designed a new peptide targeting PD-1, FITC-YT-16, which significantly enhanced the anti-tumor activity of T cells in vitro^404^, while Sasikumar et al. designed the peptide NP-12 to bind PD-L1 competitively with PD-1. Moreover, NP-12 showed the same efficacy as commercial PD-1 targeted antibodies in inhibiting the growth and metastasis of primary tumors in preclinical models of melanoma, colon cancer, and renal cell carcinoma^405^. Although these peptides are not yet suitable for blocking PD-1/PD-L1 to treat tumors, they offer promising potential. Toxic peptides (VPs) from animals may also show an anti-tumor effect. Because VPs naturally target mammalian receptors, they show a high degree of specificity and selectivity for specific ion channels and receptors on the cell membrane. Hanatoxin-1, a peptide toxin isolated from Chilean spiders, specifically blocks the K^+^ channel on the membrane^406^. High expression of the K^+^ channel has been observed during the development of colon cancer, and Okada et al. found that the porogenic peptide LaFr26 purified from *Lachesana sp*. spider venom had a cytotoxic effect on the lung cancer cell lines LX22 and BEN, which expressed an endogenous K^+^ current^407^. Attention has also been paid to the role of AMPs in tumors^408^. Some AMPs have demonstrated anti-tumor activity, while others promoted tumor development^409^. The simplified θ-defensin analogue synthesized by Strzelecka et al. inhibited the growth of breast cancer cells in a 3D culture model, indicating that θ-defensin derivatives have anticancer potential^410^. Anticancer peptides are cationic amphiphilic molecules that preferentially kill cancer cells through folding-dependent membrane rupture. Referring to the membrane-specific interaction of anticancer peptides, Aronson et al. prepared a new class of peptide lipid particles that fuse rapidly with the tumor cell membrane and mediate cell killing, with little toxicity to normal cells, indicating a new tumor-lysis strategy^411^.

Although many peptides have shown the promising anti-tumor effects in preclinical and clinical studies. Only two peptides are currently approved for the treatment of tumors, mifamurtide for osteosarcoma and carfilzomib for multiple myeloma, and research into treatment strategies involving therapeutic peptides for more common tumors, such as lung cancer and gastric cancer, is still ongoing. The key is thus to identify more receptor targets that are specifically expressed in tumor cells and to strengthen their medical translation. In addition, the combination of peptides targeting various tumor receptors is also a potential strategy.

### Antiviral peptides

Viruses parasitize all living creatures, including humans, animals, plants, bacteria, and archaea. Humans have always suffered from viral diseases, including Ebola hemorrhagic fever, influenza, and acquired immune deficiency syndrome (AIDS)^412,413^. Despite extensive efforts in antiviral drug development over the past two decades, leading to the approval and clinical use of multiple antiviral drugs^414,415^, there remains no effective treatment for some of these diseases such as AIDS.

Research into antiviral peptides has become a hot topic, because of the high specificity and activity of peptides^416^. Antiviral peptides act mainly by targeting the virus or its host to block infection^417,418^. Enfuvirtide, the first approved antiviral peptide, is a 36-amino acid peptide that blocks HIV infection by binding to the heptad-repeat domain of gp41 (HIV envelope protein) to prevent its fusion^419^. In 2011, the antiviral peptide drugs boceprevir and telaprevir were approved for clinical treatment of hepatitis C virus (HCV)^420,421^. They both bind to the HCV NS3/4A serine protease to inhibit protease activity, thus blocking HCV replication in the host^422^. More research on antiviral peptide drug candidates is being undertaken in pre-clinical and clinical studies, including myrcludex B against HBV and HDV^423–425^, flufirvitide against Influenza virus^426,427^, and sifuvirtide against HIV-1^428–430^.

Since 2020, the respiratory pandemic disease caused by the novel coronavirus SARS-CoV-2 has seriously disturbed people’s lives throughout the world^431^. Scientists have devoted extensive efforts to studying the mechanism of infection of COVID-19 since the start of the pandemic in early 2020, as well as searching for anti-COVID-19 treatments and drugs, including peptide drugs^432–436^. The COVID-19 genome was rapidly sequenced as an enveloped, positive single-stranded RNA coronavirus with a genome size of about 29.9 kb^437,438^, which is closely related to bat coronaviruses and the SARS-CoV virus^439,440^.

Vaccines are commonly considered as effective agents for preventing the spread of pandemic diseases. Vaccines have been approved and used in many countries, including vaccines based on mRNA^441,442^, recombinant adenoviral vectors^443,444^, and inactivated vaccines^445,446^. Peptide vaccines have certain advantages, such as high specificity, good safety, and easier production, and have thus become an active research area in development of vaccines against SARS-CoV-2^447–449^. Based on the infection mechanism, several research groups have designed and evaluated peptide vaccines against SARS-CoV-2. They used immunoinformatics technology to analyze and identify the key epitopes of B- and T-cell that specifically recognize the spike glycoprotein of SARS-CoV-2. Li et al. and Chakraborty et al. applied the natural epitope sequences as vaccine candidates against SARS-CoV-2^450,451^. While, Bhattacharya et al. and Waqas tried to construct new peptides as COVID-19 vaccine candidates based on the epitope fragments from B- and T-cell^452,453^. Similar work has also been carried out by other groups^454–456^. Herst et al. attempted to design COVID-19 peptide vaccines using the CTL peptide vaccine research platform for Ebola Zaire, and obtained a series of peptide vaccine candidates^457^. Many other studies have focused on blocking the infection process of SARS-CoV-2 using synthetic peptides or nucleotides^458–461^.

The development of antiviral peptides attracted wide attention during the COVID-19 pandemic, especially the development of peptide vaccines against SARS-CoV-2. Novel technologies, such as immunoinformatics characterization, epitope-based design, in silico identification, and molecular docking have been used expeditiously to design and identify peptide vaccine candidates. Although no peptide vaccines have yet been approved for the treatment of COVID-19, valuable experience has been gained in the development of peptide vaccines, not only against SARS-CoV-2, but also against new viruses in the future.

## Conclusion and perspective

Peptides have become a unique class of therapeutic agents in recent years as a result of their distinct biochemical characteristics and therapeutic potential. Although peptides outperform small molecules and large biologics in some aspects, they often suffer from membrane impermeability and poor stability in vivo, due to the intrinsic limitations of amino acids. Extensive research has been carried out in terms of the discovery, production, and optimization of peptide drugs, in order to overcome these drawbacks. The integration of traditional lead peptide discovery methods with novel technologies, such as rational design and phage display, provides a reliable approach for the development of effective and selective lead peptides in a short period of time. The single or combined use of chemical and biological recombination synthetic approaches allows the efficient and reliable production of synthetic peptides on large scales. These peptides can be further modified in a site-specific manner through chemical synthesis or genetic code expansion to enhance their stability and physiological activity.

Although the field of therapeutic peptides started with natural hormones, the discovery and development trends have since shifted from simply mimicking natural hormones or peptides derived from nature to the rational design of peptides with desirable biochemical and physiological activities. Major breakthroughs in molecular biology, peptide chemistry and peptide delivery technologies have allowed significant progress in the fields of peptide drug discovery, peptide production, and their therapeutic applications. More than 80 therapeutic peptides have reached the global market to date, and hundreds of peptides are undergoing preclinical studies and clinical development. These peptide drugs have been applied to a wide range of diseases, such as diabetes mellitus, cardiovascular diseases, gastrointestinal diseases, cancer, infectious diseases, and vaccine development. Considering their huge therapeutic potentials, market prospects, and economic values, we expect therapeutic peptides to continue to attract investment and research efforts and to achieve long-term success.
